# Knock Down of Plakophillin 2 Dysregulates Adhesion Pathway through Upregulation of miR200b and Alters the Mechanical Properties in Cardiac Cells

**DOI:** 10.3390/cells8121639

**Published:** 2019-12-14

**Authors:** Luca Puzzi, Daniele Borin, Priyatansh Gurha, Raffaella Lombardi, Valentina Martinelli, Marek Weiss, Laura Andolfi, Marco Lazzarino, Luisa Mestroni, Ali J. Marian, Orfeo Sbaizero

**Affiliations:** 1 Engineering and Architecture Department, University of Trieste, 34127 Trieste, Italy; luca.puzzi@gmail.com (L.P.); dborin@units.it (D.B.); 2 Centre for Cardiovascular Genetics, Institute of Molecular Medicine at the University of Texas Health Science Centre and Texas Heart Institute, Houston, TX 77030, USA; priyatansh.gurha@uth.tmc.edu (P.G.); ali.j.marian@uth.tmc.edu (A.J.M.); 3 Advanced Biomedical Sciences, Federico II University, 80138 Napoli, Italy; leila.lombardi1@gmail.com; 4 International Centre for Genetic Engineering and Biotechnology, 34149 Trieste, Italy; vmartinel72@gmail.com; 5 Institute of Physics, Faculty of Technical Physics, Poznan University of Technology, Piotrowo 3, 60965 Poznan, Poland; 6 CNR-IOM, Area Science Park, 34149 Trieste, Italy; andolfi@iom.cnr.it (L.A.); lazzarino@iom.cnr.it (M.L.); 7 Cardiovascular Institute, University of Colorado Anschutz Medical Campus, Aurora, CO 80045, USA; luisa.mestroni@cuanschutz.edu

**Keywords:** arrhythmogenic cardiomyopathy, PKP2, AFM, microRNA, cell adhesion, intercalated disk, focal adhesion

## Abstract

**Background:** Mutations in genes encoding intercalated disk/desmosome proteins, such as plakophilin 2 (PKP2), cause arrhythmogenic cardiomyopathy (ACM). Desmosomes are responsible for myocyte–myocyte attachment and maintaining mechanical integrity of the myocardium. **Methods:** We knocked down *Pkp2* in HL-1 mouse atrial cardiomyocytes (HL-1^Pkp2-shRNA^) and characterized their biomechanical properties. Gene expression was analyzed by RNA-Sequencing, microarray, and qPCR. Immunofluorescence was used to detect changes in cytoskeleton and focal adhesion. Antagomirs were used to knock down expression of selected microRNA (miR) in the rescue experiments. **Results:** Knockdown of *Pkp2* was associated with decreased cardiomyocyte stiffness and work of detachment, and increased plasticity index. Altered mechanical properties were associated with impaired actin cytoskeleton in HL-1^Pkp2-shRNA^ cells. Analysis of differentially expressed genes identified focal adhesion and actin cytoskeleton amongst the most dysregulated pathways, and miR200 family (a, b, and 429) as the most upregulated miRs in HL-1^Pkp2-shRNA^ cells. Knockdown of miR-200b but not miR-200a, miR-429, by sequence-specific shRNAs partially rescued integrin-α1 (*Itga1*) levels, actin organization, cell adhesion (on collagen), and stiffness. **Conclusions:** PKP2 deficiency alters cardiomyocytes adhesion through a mechanism that involves upregulation of miR-200b and suppression of *Itga1* expression. These findings provide new insights into the molecular basis of altered mechanosensing in ACM.

## 1. Introduction

In organs constantly challenged by mechanical stress, such as the heart, desmosomal cadherins, N-cadherins, and integrins are the key elements that maintain tissue homeostasis by coordinating cell–cell and cell-extra cellular matrix (ECM) interactions. Spatial and temporal regulation of these two types of adhesion mechanisms influences cell cytoskeletal dynamics, enabling cardiomyocytes to adapt to internal or external physical and biochemical stimuli. Despite the differences in their molecular compositions, integrins and cadherins are robustly integrated into convergent signaling pathways [1,2,3,4,5]. In addition, both are connected to common structural mechanoresponsive cytoskeletal elements, such as F-actin, microtubules, and intermediate filaments. The latter structures, in turn, are responsible for correct dissipation of the mechanical loads and for an efficient signal transduction, providing a scaffold for various signaling molecules activated via transmembrane receptors. Accordingly, heart tissue mechanical integrity is strictly dependent on correct cell–cell and cell–ECM connections.

Mutations in genes encoding desmosome proteins, such as *PKP2* and *DSP*, encoding plakophilin-2 (PKP2) and desmoplakin (DSP), respectively, are major causes of arrhythmogenic cardiomyopathy (ACM) [6]. Desmosomes are members of the intercalated discs (IDs) and responsible for maintaining mechanical integrity of the myocardium. ACM is a hereditary cardiomyopathy characterized by preponderance of cardiac arrhythmias occurring early and mostly independent of cardiac dysfunction [6]. In addition, fibro-fatty replacement of the myocardium is an important pathological feature of ACM. Likewise, the right ventricle is the predominant site of involvement in a subset of ACM, referred to as arrhythmogenic right ventricular cardiomyopathy (ARVC).

The mechanisms by which mutations in desmosome or intercalated disc (ID) proteins cause ACM are largely unknown. We have established PKP2-deificent HL-1 cell lines and have published the molecular phenotype in these cells, which includes activation of the mechano-sensitive Hippo and suppression of the canonical WNT pathways. We have extended the findings in the PKP2-deficient HL-1 cells to mouse models and human hearts with ACM [7,8,9]. Moreover, we have defined microRNA profile of HL-1 cells and have identified miR-200 family among the most upregulated miRs in PKP2-deficient HL-1 cells. However, the effects of these molecular changes on mechanical properties of HL-1 cells have not been explored. Given the important role of the ID/desmosome proteins in maintain mechanical integrity of cardiac cells, we posit that ACM mutations affect mechanical properties of cardiac cells. Thus, the purpose of the present study was to delineate mechanical properties of PKP2 deficient HL-1 cardiomyocytes and identify the molecular mechanisms involved.

## 2. Materials and Methods

### 2.1. Cell Culture and Immunostaining

Mouse atrial HL-1 cells (HL-1^WT^), as well as HL-1 cells deficient in PKP2 (HL-1^PKP2-shRNA^) and HL-1 cells treated with a shRNA with no known target in the genome (HL-1^NT^), were previously described [8,9]. HL-1 cell lines were cultured according to the protocol established by Dr. W.C. Claycomb [10]. Briefly, asynchronous growing cells were seeded in Petri dishes, pre-coated with 0.005% fibronectin and 0.02% gelatin, and cultured in Claycomb medium supplemented by 2 mM l-glutamine, 10% fetal bovine serum, 100 U/mL penicillin, 100 μg/mL streptomycin, and 10 mM Norepinephrine, and incubated at 37 °C and 5% CO_2_.

HL-1 cells were fixed in 4% paraformaldehyde (PFA) and were incubated with an anti-α-tubulin or an anti-vinculin primary antibody. Cells were then counterstained with an Alexa-Fluor 594 (Thermo Fisher Scientific, Waltham, MA, USA) conjugated anti mouse or anti rabbit secondary antibody. F-Actin filaments were counterstained with Alexa Fluor 594 Phalloidin and cell nuclei were counterstained with DAPI. Cells were analyzed using a Nikon (Nikon Corp., Tokyo, Japan.)

C2 Confocal Microscope System equipped with a Plan-Apochromat λ 60X/1.40 oil objective. Full description of immunostaining protocol used is provided in the Supplementary Material online.

### 2.2. Morphology and Mechanical Properties Assessment Using Atomic Force Microscopy

An AFM Solver Pro-M (NT-MDT, Moscow, Russia) was used to acquire morphology, as well as force–displacement curves, as previously reported [11,12]. sQube CP-PNPL-Au-C (Bickenbach, Germany) cantilevers were used, with a nominal spring constant of 0.08 N/m, which was checked prior to each experiment by Sader method. Measurements on single living cells were performed in physiological conditions of medium and temperature, within one hour. Since the nuclear elasticity is correlated with the stages of cell division [13], cells with nuclei optically showing mitosis were excluded. For each investigated area, a preliminary scan was made to assess the cell morphology and the nuclear position, which corresponds to the highest portion of the cell. In order to avoid possible artefacts due to substrate stiffness and/or due to hydrodynamic forces, indentations were performed above the nucleus, at the constant speed of 1 μm/s for approach and withdrawal of the cantilever.

Cell elasticity was calculated from the first portion of the indentation curve (10% of cell deformation) using the Sneddon model. All the curves analysis was performed using AtomicJ software [14]. For further details, see Supplementary Material online. To describe the cell viscoelastic behavior towards an external applied force, we used a parameter introduced by Klymenko et al. [15] and indicated as “plasticity index” *η* (even though “plastic” stands for non-recoverable deformation). This was assessed from the hysteresis between the approach and withdrawal curves (green box in Supplementary Figure S2), as:

(1) $$\eta =1-(A_{2}/A_{1})$$

where *A*_1_ and *A*_2_ are the areas under the loading and unloading curves, respectively. Intermediate values between a fully elastic (*η* = 0) and a fully plastic behavior (*η* = 1) indicate mixed viscoelastic properties.

For both the Young’s modulus and the plasticity index assessment, each cell was subjected to three consecutive indentations at the same position, and the mean of the results was considered as a single cell value (*n* = 1).

### 2.3. mRNA and microRNA Targets Analysis

Differentially expressed genes (DEGs) and microRNAs in HL-1^PKP2-shRNA^ cells were obtained from published dataset [8,9]. Pathway analysis on DEGs (*q* value > 0.05) was performed using consensus path DB (http://cpdb.molgen.mpg.de/MCPDB) and GSEA (http://software.broadinstitute.org/gsea/index.jsp). Only enriched pathways with *q* value < 0.05 were selected for presentation and further analysis. TargetScan [16], Starbase [17], and MiRWalk [18] and Ago-HITS-CLIP (argonaute high throughput sequencing after cross-linked immunoprecipitation) dataset [19] were used to predict miR targets among the DEGs. Because the miR-200 family was amongst the most upregulated miRs in the HL-1^PKP2^ cells, their targets were identified among the DEGs and then merged with the above-mentioned datasets. Pathways analysis of miR-200 targets for overrepresentation of gene set was performed using ConsensusPathDB (http://ConsensusPathDB.org).

### 2.4. Quantitative PCR

Total RNA (including miRNA) from three independent cell preparations was extracted using miRNeasy kit (Qiagen, Hilden, Germany) and cDNA was prepared using either high capacity cdna transcription kit or MicroRNA reverse transcription kit (both from Life Technology, Carlsbad, CA, USA) according to manufacturer’s protocol. Transcript levels were assessed using SYBR Green (BioRad, Hercules, CA, USA) qPCR analysis and specific primers, as described in Supplementary Material online. MiRNA levels were determined using specific TaqMan (Thermo Fisher Scientific, Waltham, MA, USA) miRNA assay. RPL37 gene and snoRNA202 values were used to normalize mRNA and miRNA expression levels, respectively. ΔΔCt method was applied to calculate the normalized gene expression values.

### 2.5. Lentiviral Vectors

Recombinant lentiviral vectors carrying shRNAs that target members of the miR-200 family (mmu-miR-200a-3p, mmu-miR-200b-3p, and mmu-miR-429-3p) and a shRNA without known target sequence in mammals (antimiR-NT) were produced using miRzip lentivector-based anti-MicroRNAs system (System Biosciences, Mountain View, CA, USA). The vectors also included a GFP reporter gene. Full description of lentiviral vector production is provided in the Supplementary Material online.

### 2.6. Cell–ECM Interaction Measured by AFM

Cell–ECM protein interaction was assessed through a JPK NanoWizard II AFM (Bruker, Berlin, Germany) equipped with a CellHesion module, using tipless V-shaped silicon nitride gold covered cantilevers having a nominal spring constant value of 0.32 N/m (NanoWorld, Innovative Technologies, Schaffhausen, Switzerland). O_2_ plasma treated cantilevers were functionalized with fibronectin (Thermo Fisher Scientific, Waltham, MA, USA)) at the final concentration of 20 µg/mL for 15 h at 4 °C, and stored in PBS [20]. Before each experiment, the cantilever spring constant was calibrated using the thermal noise method. To avoid potential ascertainment bias, cells in isolation (not attached to other cells) were selected randomly in each plate for the AFM studies. Measurements were performed according to published protocols [21,22,23]. Briefly, HL-1 cell suspension was overlaid on a BSA coated glass coverslip inserted into a petri dish previously coated with type I collagen or fibronectin (both from Thermo Fisher Scientific). A single cell from the suspension was captured on the functionalized cantilever and then pressed against the coated plastic surface at a constant force of 0.5 nN for 20 s. The work of detachment in our system was evaluated by integrating the area between the contact point on the surface and the last force interaction, which resulted in the cantilever returning to its base position. Withdrawn curves were analyzed using the JPK data processing software, classifying detachment events as “rupture” or “tether” based on the slope of the curve preceding the force step [22] (see Supplementary Figure S3). For these experiments, each cell was subjected to 4–6 consecutive indentations at the same collagen or fibronectin spot and the mean of the results was considered as a single cell–ECM interaction (*n* = 1). More details on the experiments are provided in the Supplementary Material online.

### 2.7. Statistics

All data were first subjected to the Shapiro–Wilk normality test. The unpaired one-way ANOVA with Dunnet’s or Tukey’s correction for normal distributions or the Kruskal–Wallis with Dunn’s correction test was employed. A confidence interval of 95% (α = 0.05) was used to identify significant differences among the samples. Data in the text are reported as mean of values ± standard deviation and graphically presented as scattered dot plots with mean (and median for those deviating from normality test) and standard deviation. For RT-qPCR experiments, data were presented as normalized values obtained by using the ΔΔCT method (PMID:11846609). The experiments were repeated 4–6 times. Statistical analysis was performed using GraphPad Prism software (San Diego, CA, USA). A quantification of the actin aggregates amount has been done using ImageJ (open source) software. The calculations were performed for every cell line on 20 cells from four independent experiments. More details on the experiments are provided in the Supplementary Material online.

## 3. Results

### 3.1. Knock Down of PKP-2 Affects Mechanical Properties and Morphology of HL-1 Cells

Atomic force microscopy (AFM) was used to assess the effect of PKP2 knock down on the mechanical properties of the HL-1 cells, focusing in particular on the cellular stiffness and the viscoelastic behavior. Non-transduced HL-1 cells (HL-1^WT^) and HL-1 cells transduced with a lentiviral vector carrying a non-targeting shRNA (HL-1^NT^) were included, as controls.

**Figure 1:**
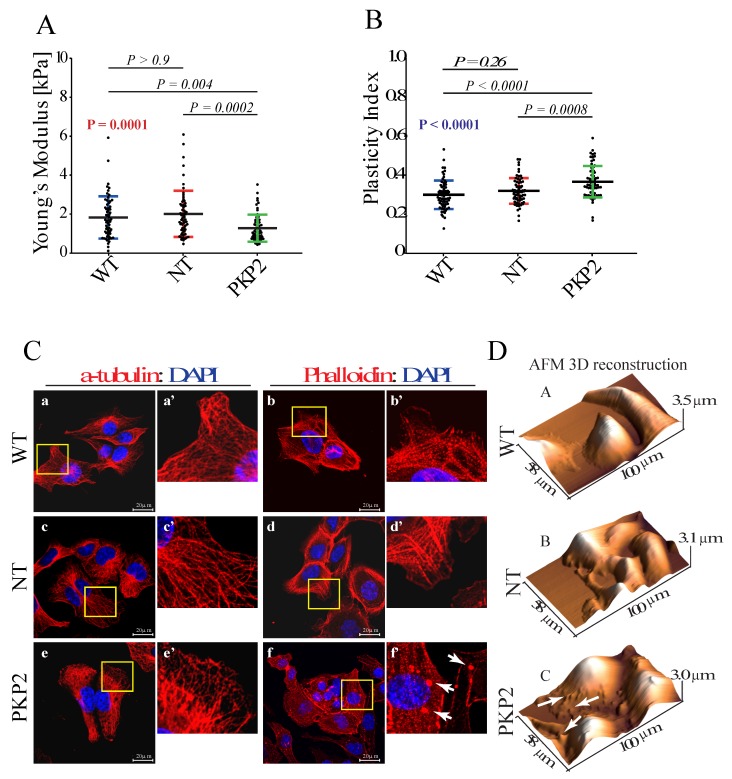
Knock down of PKP-2 affects mechanical properties and morphology of HL-1 cells. (**A**) Young’s modulus value indicated a reduced stiffness upon knockdown of PKP2 protein. HL-1^WT^: 1.65 kPa; 0.99 kPa = 25% Percentile; 2.45 kPa = 75% Percentile; HL-1^NT^: 1.74 kPa; 1.15 kPa = 25% Percentile; 2.48 kPa = 75% Percentile; HL-1^PKP2^: 1.05 kPa; 0.74 kPa = 25% Percentile; 1.57 kPa = 75% Percentile. *n* = 60 (179 force curves) for HL-1^WT^, HL-1^NT^ and (178 force curves) for HL-1^PKP2^, *n* = *n*° of cells. Kruskal–Wallis test with Dunn’s correction, *p* value in red. (**B**) Plasticity index showed that in PKP2 knockdown cells external forces are converted more into viscous losses, suggesting a compromised actin network architecture. HL-1^WT^: 0.30 ± 0.07; HL-1^NT^: 0.31 ± 0.06; HL-1^PKP2^: 0.36 ± 0.08. *n* = 68 (204 force curves) for HL-1^WT^, *n* = 67 (201 force curves) for HL-1^NT^, *n* = 69 (207 force curves) for HL-1^PKP2^. One-way ANOVA with Dunnett’s correction, ANOVA *p* value in blue. (**C**) Confocal microscopy analysis showed a conserved microtubules structure in all the conditions (panels a, a’, c, c’, e, and e’.), whereas phalloidin staining showed the presence of actin granules in PKP2 knockdown cells (e.g., white arrows in panel f’). *n* = 3 independent cell staining (scale bar 20 μm). For each IF picture high magnification panels of selected areas are shown. (**D**) AFM topographic maps confirmed the presence of actin granules, as indicated by arrows in panel c.

The Young’s modulus was used as a quantitative parameter to assess changes in cell stiffness that result from alteration of cellular tensional elements. It enables assessing physiological response of the cell to external forces, despite heterogeneity resulting from internal structure and anisotropy (each internal component differently contributing to cell elasticity and viscosity). Knock down of PKP2 was associated with decreased cellular stiffness, as indicated by decreased Young’s modulus, as compared to control groups (Figure 1A).

The effect of PKP2 knockdown on the viscoelasticity of HL-1 cells was depicted by the plasticity index ^15^, which describes the relative elastic/viscous behavior of the cells undergoing external forces. Data obtained in HL-1^PKP2-shRNA^ cells indicated that external forces are dissipated into viscous effects to a greater extent than in HL-1^WT^, due to lower cell resistance (Figure 1B). Given that visco-elastic properties of cells rely directly on cytoskeletal architecture, confocal microscopy analysis was performed to assess actin microfilaments and microtubules status. Moreover, cell surface morphology was checked using AFM scan topographic images. The results showed that loss of PKP2 protein was associated with a perturbation of the actin network, which resulted in the presence of actin aggregates (e.g., see arrows in Figure 1C, panels f and f’, and Figure 1D, panel c), while the microtubules network (assessed by α-tubulin staining) remained intact (Figure 1C panels a, a’, c, c’, and e and e’). A quantification of the actin aggregates (the amount of aggregates area on the total cytoskeleton area after Phalloidin staining) showed: (median: NT = 1.08%, WT = 0.97%, PKP2 = 2.90%, (p value calculated using unpaired Wilcoxon-Mann-Whitney test (confidence level 95%), NT vs. WT: *p*-value = 0.4652, NT vs. PKP2: *p*-value = 1.466 × 10^−7^, WT vs. PKP2: *p*-value = 1.475 × 10^−7^).

### 3.2. PKP-2 Knockdown Affects Focal Adhesion Pathway

**Figure 2:**
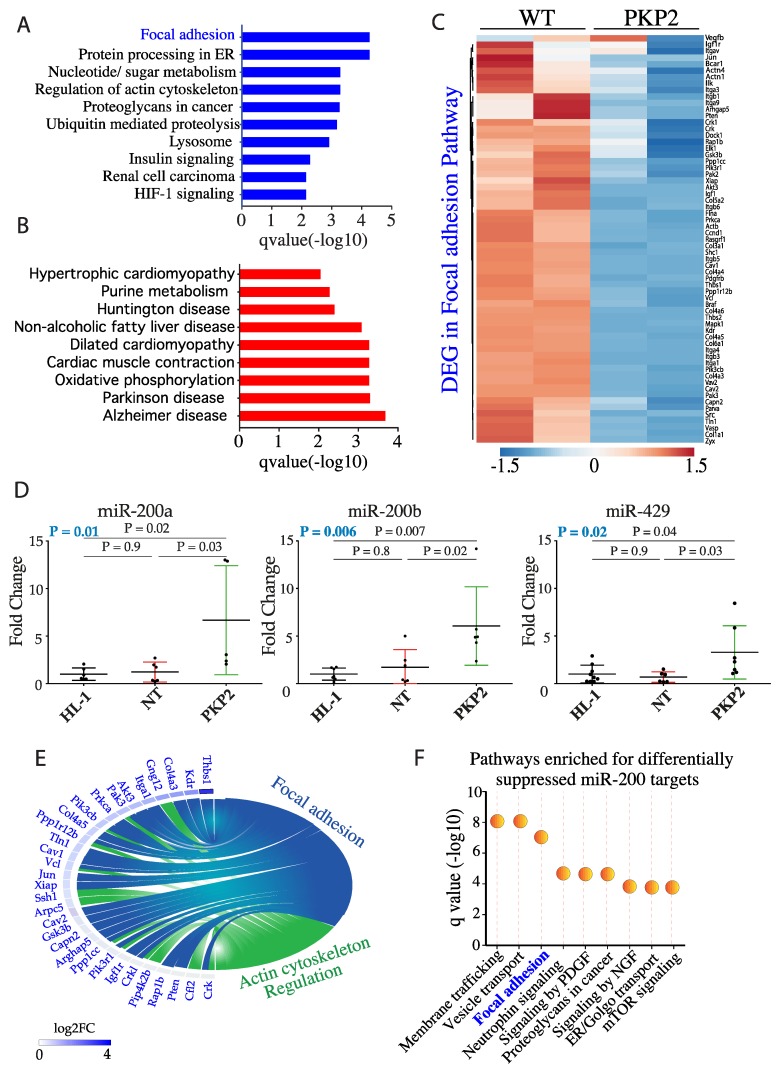
PKP-2 knockdown affects focal adhesion pathway. (**A**) Pathway analysis of genes in HL-1^PKP2^ cells showing the focal adhesion pathway as the most downregulated by PKP2 knockdown. (**B**) Pathway analysis of genes in HL-1^PKP2^ showing those involved in myocardial hypertrophy as the most upregulated. (**C**) Heat plot map showing the differentially expressed genes (DEG) involved in focal adhesion pathway due to PKP2 knockdown in HL-1 cells. (**D**) Quantitative PCR showing the upregulation of mmu-miR-200a, mmu-miR-200b and mmu-miR429 in PKP2 knockdown HL-1 cells. *n* = 6 independent experiments for miR-200a, *n* = 7 for miR-200b and miR-429; one-way ANOVA with Dunnett’s correction; ANOVA *p* value in blue. (**E**) Circle plot showing the specific target genes involved in focal adhesion (blue) and actin cytoskeleton regulation (green). (**F**) Pathway analysis of miR-200 targets indicates the focal adhesion as one of the most dysregulated pathways.

To identify molecular pathways responsible for altered mechanical properties of HL-1^PKP2^ cells, the RNA-Seq data were analyzed to identify DEGs and the corresponding dysregulated biological pathways [8]. Genes involved in the focal adhesion pathway, responsible for transmitting mechanical force between the cell and the extracellular matrix (ECM), were amongst the most downregulated (Figure 2A, −log_10_ *q* = 4.27), whereas those involved in myocardial hypertrophy and function were the most upregulated genes (Figure 2B). The list of DEGs included 63 genes (of 200 in the KEGG database) that were part of the focal adhesion pathway and were suppressed in the HL-1^PKP2^ cells (Figure 2C).

### 3.3. DEGs in the Focal Adhesion Pathways are Targets of miR200b

Given that miR-200 family were amongst the most dysregulated miR in HL-1^PKP2^ cells [9], transcript levels of each member of miR200 family were quantified in the in HL-1^PKP2^ and control cells by qPCR. Consistent with previous observation [9], RT-qPCR analysis showed that three members of miR200, namely mmu-miR-200a-3p, mmu-miR-200b-3p, and mmu-miR429-3p, were markedly upregulated in HL-1^PKP2^ cells (Figure 2D). Other miR200 family members, namely miR-200c and miR-141, were not differentially expressed [9] and therefore not analyzed here. To determine the effects of upregulation of miRs on their targets, DEGs were analyzed to identify the putative targets of miR-200 family by TargetScan [16], Starbase [17], and MiRWalk [18] and Ago-HITS CLIP datasets. A total of 816 DEGs in the HL-1^PKP2-shRNA^ cells were identified putative miR-200 targets. Expression levels of 560/816 (69%) putative miR-200 target genes were reduced in the HL-1^PKP2-shRNA^ cells (*p* < 0.001, Supplementary Table S1). Gene set over-representation analysis of the downregulated targets showed enrichment for genes involved in focal adhesion (27 genes, *q* = 9.2 × 10^−8^) and actin cytoskeleton organization (18 genes, *q* = 0.0006) as the top dysregulated pathways (Figure 2E,F; Table S2).

**Figure 3:**
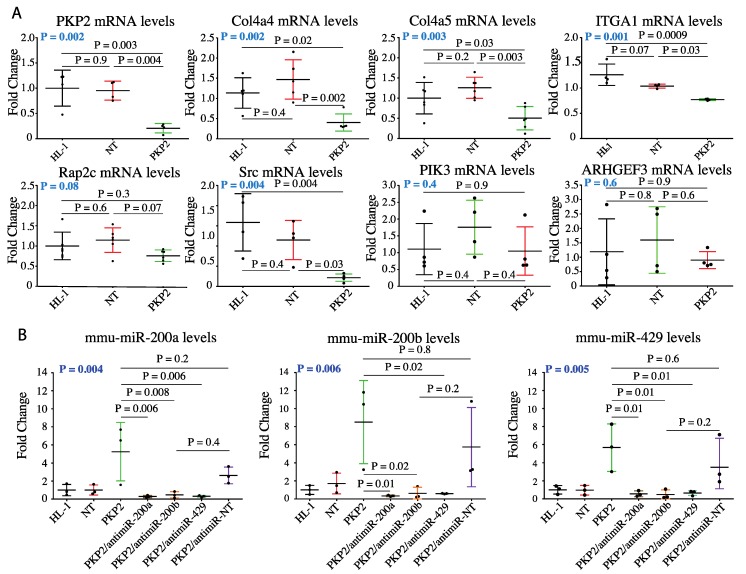

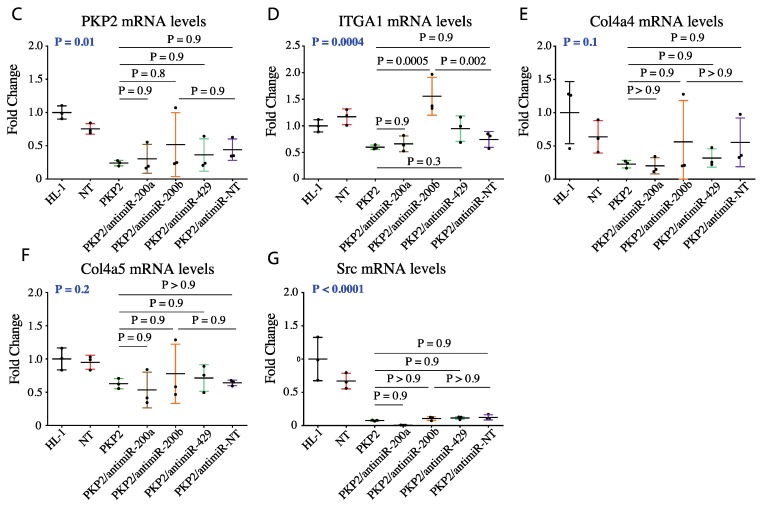
miR200 target analysis and Itga1 rescue by antimiR-200b. (**A**) qPCR analysis of the expression levels of selected miR-200 targets that are part of focal adhesion pathway confirmed Col4a4, Col4a5, Src, and Itga1 as miR-200 family targets, which are downregulated in PKP2 knockdown cells. Pkp2 mRNA expression analysis was included as a control. *n* = 5 for Col4a4, Col4a5, and *n* = 4 for Pkp2 (as a control), Itga1, Rap2c, Src, Arhgef3, and Pik3cb independent experiments were done, respectively. One way-ANOVA with Dunnett’s correction; bars represent mean ± standard deviation. ANOVA P value in blue. (**B**) qPCR analysis of microRNA expression revealed that stable expression of antagomiR-200a and antimiR-200b in PKP2 knockdown cells restored expression of all the miR-200 family members. *n* = 3 independent experiments were done for all stable cell lines. One-way ANOVA with Tukey’s correction; bars represent mean ± standard deviation. ANOVA *p* value in blue. (**C**–**G**) Gene expression of the four miR-200 family target genes previously found to be dysregulated upon PKP2 knockdown was assessed by qPCR in cells stably expressing each antimiR. The analysis revealed restored expression levels of Itga1 after downregulation of miR-200b (**D**), while the levels of the other targets were unchanged (**E**–**G**). miR-200a and miR-429 had no significant effects on the transcript levels of selected gene members of the focal adhesion pathway (**D**–**G**). Pkp2 mRNA expression analysis was included as a control (**C**). *n* = 3 independent experiments were done for all stable cell lines. One way-ANOVA with Tukey’s correction; bars represent mean ± standard deviation. ANOVA *p* value is in blue.

To validate the findings on miR-200 family target genes, seven candidates that were members of the cell adhesion pathway were validated by qPCR. The selected genes coded for the ECM binding protein ITGA1 (Integrin Subunit Alpha-1), the downstream signaling transductors SRC (proto-oncogene tyrosine-protein kinase Src), PIK3CB (Phosphatidylinositol 4,5-bisphosphate 3-kinase catalytic subunit beta isoform), the transductor assistant ARHGEF3 (Rho guanine nucleotide exchange factor 3), the collagen extracellular matrix constituents COL4A4 (Collagen type IV alpha-4 chain), COL4A5 (Collagen type IV alpha-5 chain), and the cell–cell junction component RAP2C (Ras-related protein Rap-2c). Among the selected targets, transcript levels of *Col4a4*, *Col4a5*, *Itga1*, and *Src* were significantly downregulated in the HL-1^PKP2-shRNA^, as compared with HL-1^WT^ and HL-1^NT^ cells (Figure 3A).

### 3.4. Knock Down of miR200b Rescued Itga1 Levels

To determine whether the miR-200 family directly regulated candidate cell adhesion molecules, expression levels of miR-200a, miR-200b, and miR-429 were knocked down using specific antagomiRs. Stable HL-1^PKP2-shRNA^ cell lines expressing antagomiRs targeting each component of the miR-200 family were generated upon transduction with recombinant lentiviral vectors (HL-1^PKP2/antimiR-200a^, HL-1^PKP2/antimiR-200b^ and HL-1^PKP2/antimiR-429^). Cells transduced with a non -targeting antagomiR lentiviral vector was used as a control (HL-1^PKP2/antimiR-NT^), along with non-transduced HL-1 cells. Quantitative analysis of miR-200a, miR-200b, and miR-429 levels by Taqman qPCR confirmed their downregulations in the HL-1 cells transduced with the corresponding anti-miRNA viruses (Figure 3B). To determine whether targeting miR-200 family members affected *Pkp2* expression, *Pkp2* levels were determined by qPCR in the HL-1^PKP2-shRNA^ cells upon knock down of each miR-200 family member. Knock down of miR200 family did not affect *Pkp2* transcript levels in HL-1^PKP2-shRNA^ cells (Figure 3C).

Next, we determined effects of knock down of miR-200a, miR-200b, and miR-429 on the transcript levels of selected dysregulated molecules in the focal adhesion pathway predicted to be targets of miR200 family, namely *Itga1*, *Col4a4*, *Col4a5*, and *Src* in HL-1^PKP2^ cells. Only suppression of miR-200b, but not miR-200a or miR-429 levels, restored *Itga1* expression levels (Figure 3D) without a significant effect on *Col4a4*, *Col4a5*, and *Src* levels (Figure 3 E, F, and G, respectively). In addition, neither miR-200a nor miR-429 had any significant effect on the transcript levels of selected gene members of the focal adhesion pathway (Figure 3D–G).

### 3.5. Downregulation of miR-200b Partially Rescues Impaired Mechanical Properties of PKP-2 Deficient HL-1 Cells

**Figure 4:**
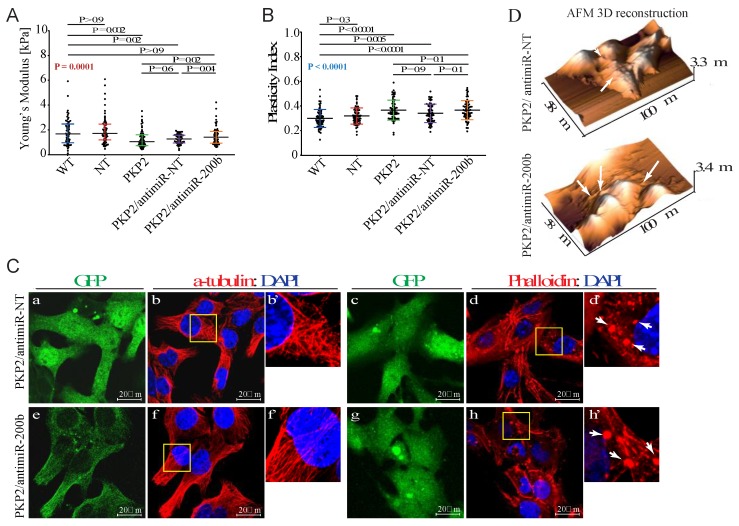
Downregulation of miR-200b partially rescues mechanical properties of PKP-2 deficient HL-1 cells. (**A**) Downregulation of miR-200b induces a rescue of the Young’s modulus value in PKP2 knockdown cells. HL-1^WT^: 1.65 kPa; 0.99 kPa = 25% Percentile; 2.45 kPa = 75% Percentile; HL-1^NT^: 1.74 kPa; 1.15 kPa = 25% Percentile; 2.48 kPa = 75% Percentile; HL-1^PKP2^: 1.05 kPa; 0.74 kPa = 25% Percentile; 1.57 kPa = 75%; HL-1^PKP2/antimiR-NT^: 1.24 kPa; 0.94 kPa = 25% Percentile; 1.56 kPa = 75% Percentile; and HL-1^PKP2/antimiR-200b^: 1.40 kPa; 0.92 kPa = 25% Percentile; 1.87 kPa = 75% Percentile. *n* = 60 (179 force curves) for HL-1^WT^, HL-1^NT^, and (178 force curves) HL-1^PKP2^, *n* = 56 (166 force curves) for HL-1^PKP2/antimiR-NT^ and *n* = 53 (159 force curves) for HL-1^PKP2/antimiR−200^, *n* = *n*° of cells. Kruskal–Wallis test with Dunn’s correction *p* value in red. (**B**) Plasticity index showed that the presence of antagomiR-200b does not rescue the viscoelastic properties in PKP2 knockdown cells. HL-1^WT^: 0.30 ± 0.07; HL-1^NT^: 0.31 ± 0.06; HL-1^PKP2^: 0.36 ± 0.08; HL-1^PKP2/antimiR-NT^: 0.34 ± 0.07, and HL-1^PKP2/antimiR-200b^: 0.36 ± 0.07. *n* = 68 (204 force curves) for HL-1^WT^, *n* = 67 (201 force curves) for HL-1^NT^, and HL-1^PKP2/antimiR-NT^, *n* = 69 (207 force curves) for HL-1^PKP2^, and HL-1^PKP2/antimiR-200b^. One-way ANOVA with Dunnett’s correction. ANOVA *p* value in blue. (**C**) Confocal microscopy analysis showed a conserved microtubules structure after antimiR expression (panels b, b’, f, and f’). Phalloidin staining showed the partial recovery of F-actin by antimiR-200b expression, whereas actin granules were still present (white arrows in panels d, d’, h, and h’). GFP channel (panels a, c, e, and g) was used as antagomiRs transduction control. *n* = 3 independent cell staining (scale bar 20 μm). (**D**) AFM topographic images confirmed the presence of actin granules also after antimiR-200b expression (arrows in panels).

Integrin-based adhesion is pivotal for translating force transmission between cells and the extracellular matrix (mechanosensing) into biological information (mechanotransmission) which, in turn, elicits a series of dynamic signaling events (mechanotransduction) [24]. To determine effects of miR-200b on cellular stiffness and viscous behavior of HL-1^PKP2-shRNA^ cells, miR-200b was knocked down by sequence-specific antagomir in these cells followed by assessment of the mechanical properties using AFM. The AFM findings are notable for recovery of the Young’s modulus values, upon knock down of miR-200b, as compared to HL-1^PKP2-shRNA^ cells (Figure 4A). However, the plasticity index was not restored upon transduction with the antagomiR-200b vector (Figure 4B). Immunofluorescence analysis showed partial rescue of F-actin network, since actin granules, while reduced, were still visible after the antimiR-200b expression in the HL-1^PKP2-shRNA^ cells (e.g., arrows in Figure 4 panels d’ and h’, and Figure 4D). A quantification of the actin aggregates showed medians: PKP2 = 2.90%, PKP2/antimiR = 2.59% (PKP2 vs. antimiR: *p*-value = 0.04873). Taken together, the AFM and immunofluorescence data indicated the presence of additional factors regulating the cytoskeletal organization, which are not affected by up- or down-regulation of miR-200b.

**Figure 5:**
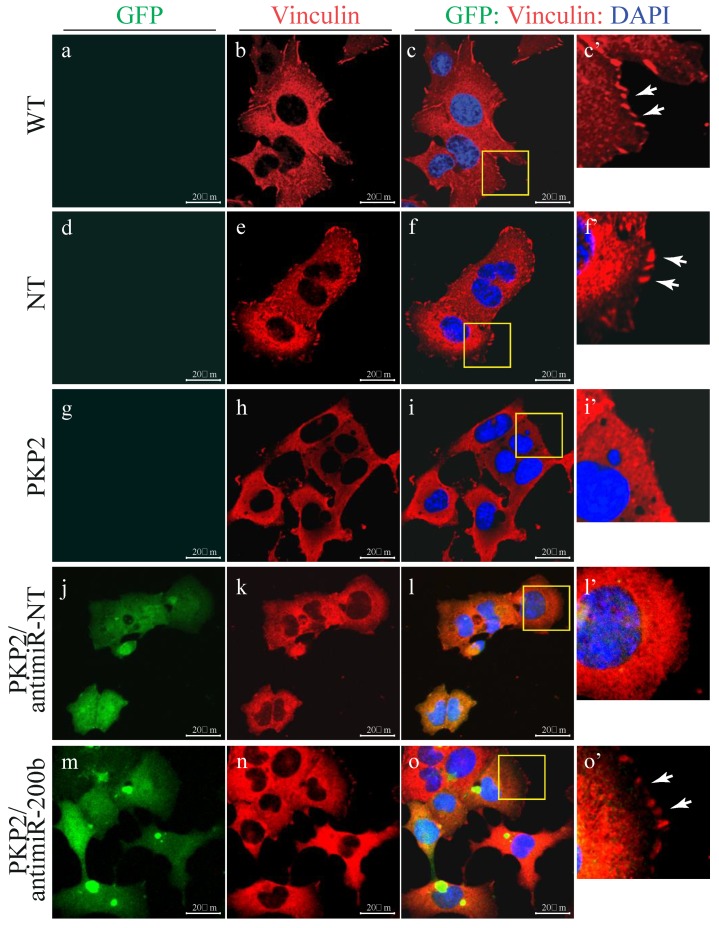
Confocal microscopy analysis of Vinculin showing that miR-200b inhibition restored focal adhesion organization. Indirect immunostaining of Vinculin (target of miR-200b and one of the first molecular sensors of external mechanical stimuli, connecting actin with the focal adhesion plaques) showed the classical “finger shape” of the focal adhesion complex in HL1^WT^ and HL1^NT^(e.g., white arrows in panels c’–f’). This phenotype was not visible in PKP2 knockdown cells, indicating disruption of the focal complex organization (panel i’). The normal phenotype was fully restored upon miR-200b inhibitor transduction (e.g., white arrows in panel o’), while no effect was observed after a no-target antimiR (panel l’) highlighting the desmosome-focal adhesion interconnection through miR-200b. *n* = 3 independent cell staining; scale bar 20 μm. High magnification panels of selected areas are shown.

Given that knock down of PKP2 was associated with altered actin network, to further support the findings, localization, and distribution of the actin-binding protein Vinculin (VCL) was analyzed by confocal microscopy (Figure 5). VCL is known to anchor and promote assembly of the actin cytoskeleton to the cell membrane through either integrin-containing focal adhesions [25] or cadherin and catenin-containing adherents junctions [26]. In support of the hypothesis of dysregulation of the actin-interacting proteins in our model, RNA-seq analysis indicated that *Pkp2* knockdown was associated with a reduced expression of vinculin (−1.74 fold change compared to wild type HL-1) which is also a target gene of miR-200b. Vinculin was checked for its morphological aspect by immuno-staining. PKP2 downregulation was associated with loss of the classical “finger shape” morphology visible in the wild type and no-target HL-1 cells (e.g., white arrows in Figure 5, panels c’ and f’), suggestive of focal adhesion disorganization. Downregulation of miR-200b by antagomiR-200b fully rescued VCL organization in the HL-1^PKP2-shRNA^ cells (e.g., white arrows in Figure 5, panel o’).

### 3.6. Downregulation of miR-200b Rescues Cell–ECM Adhesion in the HL-1^PKP2-shRNA^ Cells

**Figure 6:**
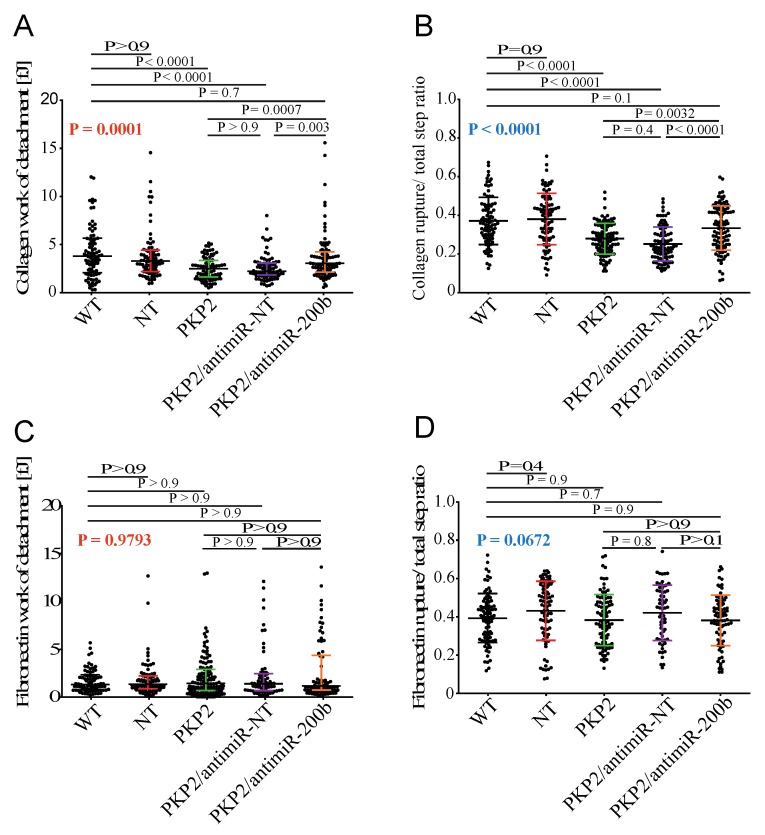
miR-200b inhibition leads to a recovery of the cell–ECM adhesive interactions detected by AFM. (**A**) The analysis of the work of detachment on type I collagen highlighted a consistent cell adhesion decrease in PKP2 deficient cells, which is completely restored by miR-200b downregulation. HL-1^WT^: 3.77 kPa; 2.07 kPa = 25% Percentile; 5.54 kPa = 75% Percentile; HL-1^NT^: 3.24 kPa; 2.22 kPa = 25% Percentile; 4.34 kPa = 75% Percentile; HL-1^PKP2^: 2.46 kPa; 1.54 kPa = 25% Percentile; 3.33 kPa = 75%; HL-1^PKP2/antimiR-NT^: 2.12 kPa; 1.78 kPa = 25% Percentile; 3.04 kPa = 75% Percentile; and HL-1^PKP2/antimiR-200b^: 2.95 kPa; 2.06 kPa = 25% Percentile; 4.22 kPa = 75% Percentile. *n* = 60 (179 force curves) for HL-1^WT^, HL-1^NT^, and (178 force curves) HL-1^PKP2^, *n* = 56 (166 force curves) for HL-1^PKP2/antimiR-NT^, and *n* = 53 (159 force curves) for HL-1^PKP2/antimiR−200^, *n* = *n*° of cells. Kruskal–Wallis test with Dunn’s correction. *p* value in red. (**B**) Ruptures versus total step ratio analysis by AFM confirmed the impaired adhesion of PKP2 knockdown cells on collagen, which is restored by antimiR-200b expression. HL-1^WT^: 0.37 ± 0.12; HL-1^NT^: 0.38 ± 0.13; HL-1^PKP2^: 0.28 ± 0.08; HL-1^PKP2/antimiR-NT^: 0.25 ± 0.09 and HL-1^PKP2/antimiR-200b^: 0.33 ± 0.11. *n* = 85 force curves for HL-1^WT^, *n* = 84 force curves for HL-1^NT^, *n* = 86 force curves for HL-1^PKP2^, *n* = 87 force curves for HL-1^PKP2/antimiR-NT^, and *n* = 89 force curves for HL-1^PKP2/antimiR-200b^ different collagen spots. One-way ANOVA with Tukey’s correction. ANOVA *p* value in blue. (**C**) work of detachment HL-1^WT^: 1.35 kPa; 0.78 kPa = 25% Percentile; 2.21 kPa = 75% Percentile; HL-1^NT^: 1.31 kPa; 0.87 kPa = 25% Percentile; 2.18 kPa = 75% Percentile; HL-1^PKP2^: 1.44 kPa; 0.72 kPa = 25% Percentile; 2.85 kPa = 75%; HL-1^PKP2/antimiR-NT^: 1.41 kPa; 0.73 kPa = 25% Percentile; 2.45 kPa = 75% Percentile; and HL-1^PKP2/antimiR-200b^: 1.12 kPa; 0.70 kPa = 25% Percentile; 4.32 kPa = 75% Percentile. Kruskal–Wallis test with Dunn’s correction. *p* value in red and (**D**) ruptures versus total steps ratio on fibronectin did not show any adhesion variation. One-way ANOVA with Tukey’s correction. ANOVA *p* value in blue. Adhesion work: *n* = 81 force curves for HL-1^WT^ and HL-1^PKP2/antimiR-200b^, *n* = 84 force curves for HL-1^NT^, *n* = 100 force curves for HL-1^PKP2^, and *n* = 69 force curves for HL-1^PKP2/antimiR-NT^ different fibronectin spots. Kruskal–Wallis test with Dunn’s correction, *p* value in red. Fibronectin cell–ECM: *n* = 87 force curves for HL-1^WT^, *n* = 83 force curves for HL-1^NT^, *n* = 94 force curves for HL-1^PKP2^, *n* = 63 force curves for HL-1^PKP2/antimiR-NT^, and *n* = 80 force curves for HL-1^PKP2/antimiR-200b^ different fibronectin spots. For all experiments, data were collected from 15 independent cells for each condition and each cell was tested 4–6 times for each ECM spot.

Given the effects of PKP2 knockdown on the miR-200/integrin-α1 axis, we assessed interaction of HL-1 cells with ECM using single cell force spectroscopy (SCFS) by AFM [22,23] (Figure S3). Among the extracellular matrix components with a known structural function in the heart, we focused on type I collagen because it is a binding substrate of integrin-α1, whereas we used fibronectin as a control. Consistent with the role of PKP2 in cell adhesion, knockdown of PKP2 led to reduced interaction of HL-1 cells with collagen, expressed as work of detachment, i.e., the energy required to detach the cell from the substrate. Interestingly, knock down of miR-200b by antagomir-200b restored collagen work of detachment in the HL-1^PKP2-shRNA^ cells (Figure 6A). Similarly, PKP2 downregulation was associated with a reduced ratio of collagen rupture to total steps, which was also restored upon suppression of miR-200b levels in HL-1^PKP2-shRNA^ cells (Figure 6B). The adhesion steps in the retracting AFM curve, corresponding to cell–ECM interactions, are considered to calculate the ratio between cell–ECM ruptures (signals due to cell membrane adhesion receptors linked to the cytoskeleton, such as integrins) and global steps number, including tethers (signals due to receptors not anchored to the cytoskeleton) (see Figure S3) [22,23]. In contrast, no notable changes were observed in the work of detachment when cells were probed over fibronectin substrate (Figure 6C). Similarly, no noticeable changes were observed in the rupture versus total steps ratio when probing cells over fibronectin substrate (Figure 6D). The findings are in accord with the data identifying *Itga1* as a target of miR-200b. Moreover, the complete recovery of the cell–collagen adhesion, and the increased ratio of ruptures detected (related to cell membrane proteins connected to the cytoskeleton) upon suppression of miR-200b, strongly support the role of antimiR-200b in rescuing the cell–ECM adhesion in part through normalization of the integrin-α1 protein expression levels.

## 4. Discussion

The mechanisms governing the pathogenesis of arrhythmogenic cardiomyopathy (ACM), a genetic disorder mostly caused by mutations in desmosome genes, are still elusive. In this study, we report that deficiency of PKP2, the most common ACM gene, alters adhesion of HL-1 cardiomyocytes to the extracellular matrix (ECM). Our findings indicate a novel interconnection between two adhesome components, PKP2 and ITGA1. We show that PKP2 deficiency is associated with upregulation of the miR-200 family, specifically miR-200b, which in turn suppresses *Itga1* expression, an important determinant of ITGA1-mediated cell–ECM adhesion in cardiomyocytes. Furthermore, we report that PKP2 downregulation leads to cytoskeletal architecture impairment, which, together with the lack of adhesion, dramatically alter biomechanical properties of the cell.

In the heart, both desmosomes/IDs and focal adhesion plaques are directly connected to structural tension elements, intermediate filaments, and F-actin, which are the main structures responsible for dissipating three-dimensional forces generated during cardiac contraction. Consequently, downregulation of PKP2 would be expected to affect adhesive structures, such as the integrin-based cell–substrate junctions.

According to the tensegrity model [27,28,29], mechanical properties of adherent cells depend on the correct balance between internal pre-existing tensional stresses, generated by F-actin and transmitted over intermediate filaments, and resistance elements, namely cytoskeletal microtubules and cell–ECM interactions through the transmembrane focal adhesion complex [29]. If this intricate and delicate balance between structural cell components is maintained, under physiological conditions, external forces transmitted through the cell can be either balanced or dissipated by mechanosensitive proteins, such as actin. The presence of actin aggregates is symptomatic of a deregulated balance leading to the deformation (buckling) of microtubules as the only way to convert the mechanical loads. This is reflected in a reduced Young’s Modulus and increased plasticity index for HL-1^PKP2-shRNA^ cells. Actin aggregation, typically resulting from misfolded proteins, is a hallmark of several diseases, including proteotoxic cardiomyopathies [30]. Actin aggregates are found in the brain neurons of patients suffering from neurodegenerative disorders [31]. In the cardiomyocyte-specific *Wdr1* deletion model, actin aggregates are associated with a high rate of postnatal sudden mice death [32]. Other cytoskeletal protein aggregates have been described in cardiomyopathies: in desmin-related cardiomyopathy, desmin, an intermediate filament, forms aggregates in cardiomyocytes [33]. Finally, in a PKP2 knock-out mouse model, Grossmann et al. [34] described dissociation of desmoplakin from the adherent junctions, and formation of granular aggregates in cardiomyocytes. Therefore, the finding of cytoskeleton protein aggregates in the present study is in line with previous studies implicating protein aggregation in the pathogenesis of cardiomyopathies. These altered mechanical properties might provide a basis for activation of the mechano-sensitive Hippo pathway and suppression of gene expression through YAP-TEAD transcriptional machinery, as previously reported [8]. Desmosomes and cytoskeletal actin are known to interact with intermediate filaments (IFs), which are also involved in mechano-transduction. Among the IFs, lamin A/C (LMNA) are mainly responsible for the nucleus mechanical integrity. Since cellular prestresses govern nuclear deformation through F–actin/lamin interaction, and since altered actin network is responsible for nuclear distortion, both aberrant nuclear shape and reduced elastic modulus could be explained by RNA-seq data reported previously, indicating that PKP2 knockdown leads to a downregulation of laminA/C gene (−1.6 fold change in HL-1^PKP2^ compared to wild type cells), which was already demonstrated as accountable for enhanced nuclear deformability. The findings, however, are not sufficient to exclude potential contributions of the IFs to altered mechanical properties of HL-1^Pkp2-shRNA^ cells. The extracellular matrix controls cytoplasmic architecture, and hence cellular mechanics, through the bidirectional “inside-out” mechanosensing signals generated by integrins-ECM binding [35]. Rescue of *Itga1* expression and failure to restore actin network upon downregulation of miR-200b suggests the presence of several unidirectional control levels of the mechanotransduction signaling. In this context, miR-200b regulated only *Itga1* expression, and consequently affected only cell–ECM adhesion. Functional changes are likely reflective of collective effects of multiple molecules and are not necessarily exclusive to the effects of rescue of Itga1 expression. Therefore, our findings do not exclude contributions of additional targets of the miR-200 family in altering the transcriptional and mechanical properties of HL-1^PKP2-shRNA^ cells. Nevertheless, they emphasize the important role of microRNAs in regulating cell function. This is particularly relevant considering that microRNA have gained tremendous attention as biomarkers and targets for novel therapeutics in the clinical setting, particularly in cardiomyopathies and heart failure [36].

### Study Limitations

Our studies were focused on cell–ECM interactions and did not include cell–cell attachment. The latter is a distinct function of the intercalated disc proteins, particularly desmoglein-2 (DSG2) and desmocollin-2 (DSC2), as well as adherens junction proteins, which span the cell cytoplasmic membrane and attach the adjacent cells together. Intercalated disc proteins PKP2, along with DSP and junction protein plakoglobin, anchor to the cell membrane and interact with cytoplasmic intermediate filaments and cytoskeleton proteins. Furthermore, miR-200 family has been shown to regulate cell–ECM interactions [19,37,38], which was also observed in the present study in HL-1^PKP2^ mouse cardiac cell model. Nevertheless, PKP2 is expected to have structural and functional effects on cell–cell attachment, which were not explored in the present study. The latter is a limitation of AFM, which enables assessment of mechanical properties of individual cells but not best suited for the assessment of cell–cell attachments. Additional experiments using different sets of tools would be necessary to assess the effects of deficiency of PKP2 on cell–cell interactions.

The findings in HL-1 cells, which are transformed atrial myocytes, might not reflect the mechanisms operational in ventricular myocytes in ACM [10]. Given that ACM, at the clinical level, predominantly involves the ventricles, studies in ventricular myocytes might provide more pertinent insights. Nevertheless, atrial tissue also expresses desmosome proteins and is commonly affected in ACM, as indicated by the presence of supraventricular arrhythmias, including atrial fibrillation PMID: 31387820, 29897149, and 31310380. In addition, human ventricular myocytes from patients with PKP2 mutations are not easily available to conduct similar studies.

The effect size of miR-200b antagomir on rescuing biomechanical properties of HL-1^PKP2-shRNA^ cells was relatively modest, which is largely in accord with multifarity of the factors involved in the pathogenesis of the phenotype. The model used for estimating the Young’s modulus value is conventionally used to yield a general idea of cell elasticity [39]. Other models could be used for this task. One fairly new and accurate is the “Brush Model” developed by Sokolov et al. [40,41,42], which can be useful to isolate the mechanical response of the cell from deformation of the pericellular layer surrounding the cell itself. The model used in the present study does not consider the brush but was chosen as (i) it is the most commonly used model, (ii) the same protocol/methodology has been used for all cells and indentations. Therefore, it has been considered that the calculated Young’s modulus values can be used to compare all cells within the presented experiments. Furthermore, resulting Young’s modulus data are reasonably similar to others reported by another group for the same cell line [43].

## 5. Conclusions

Deficiency of PKP2, as observed in ACM, leads to upregulation of the microRNA-200 family, in particular miR-200b, in cardiomyocytes, which targets the cell adhesion molecule *Itga1*, leading to impaired cytoskeleton, adhesion, and mechanical properties of the cells. Our findings provide new mechanistic insights into the altered mechanotransduction/mechanosensing in ACM [8], and sets the stage for further investigations to determine if modulation of expression of focal adhesion targets by anti-microRNA could be a viable treatment strategy [36].

## Supplementary Materials

The following are available online at https://www.mdpi.com/2073-4409/8/12/1639/s1. Supplementary Materials contain in-depth information on the materials and methods used moreover, a summary of the genes altered by PKP2 downregulation is reported.
