# Supplementary material for: Knock Down of Plakophillin 2 Dysregulates Adhesion Pathway through Upregulation of miR200b and Alters the Mechanical Properties in Cardiac Cells
Source: Cells. 2019 Dec 14;8(12):1639. doi: 10.3390/cells8121639 (PMC6952926; doi:10.3390/cells8121639)
Supplement: Supplementary file 1 [file cells-08-01639-s001.pdf]

Knock Down of Plakophillin 2 Dysregulates Adhesion Pathway through Upregulation of miR200b and Alters the Mechanical Properties in Cardiac Cells

Puzzi L.[§], Borin D.[§], Gurha P., Lombardi R., Martinelli V., Weiss M., Andolfi L., Lazzarino M., Mestroni L., Marian A.J. and Sbaizero O*.

§ = share first authorship

*** Corresponding Author**

Orfeo Sbaizero, PhD.; Engineering and Architecture Department; University of Trieste; Via Valerio 10; 34127 Trieste (Italy). Phone (+39)040-5583770; fax: (+39)040572044; Email: sbaizero@units.it

Lentiviral vector production, cell culture and immunofluorescence analysis: The lentiviruses were produced by transfecting the plasmids into 293T cells using X-tremeGENE9 DNA transfection reagent (Roche Life Science) and the viral particles were harvested at 48 and 72 hrs post-transfection, cleared by precipitation and finally filtered through 0.45 µm-pore cellulose acetate filters (WWR International). 70-90% confluent HL-1^{PKP2} cells were subjected to 48 hrs of virus infection and then cultured in virus-free medium for another 24 hrs; GFP signal was used to assess transduction efficiency by both fluorescence microscopy and FACS. GFP positive cells were isolated by flow cytometry. Asynchronous growing HL-1 cells were seeded in Petri dishes pre-coated with 0.005% fibronectin and 0.02% gelatin and cultured in Claycomb medium supplemented by 2 mM L-glutamine, 10% of fetal bovine serum, 100U/ml penicillin, 100 µg/ml streptomycin and 10 mM Norepinephrine and incubated at 37° C and 5% CO2. All chemicals for cell culture were purchased from Sigma-Aldrich.

Immunostaining of HL_1 cells was performed by first washing the cells twice with PBS and fixing with 4% PFA at room temperature for 20 minutes; thereafter aldehydes were quenched with 0.1 M glycine for 20 minutes at room temperature. Cells were made permeable with 0.5% Triton X-

100 for 10 minutes, blocked with 1:5 goat serum in PBS for 1 h at room temperature and incubated with an anti α-tubulin (mouse monoclonal Sigma Aldrich T5168, dilution 1:4000) or an anti Vinculin (mouse monoclonal Sigma Aldrich V9131, dilution 1:400) primary antibody diluted in blocking solution overnight at 4°C. Then, cells were washed 3 times with 0.5% Triton X-100 and finally incubated with an Alexa-Fluor 594 conjugated anti mouse or anti rabbit secondary antibody (Thermo Fisher Scientific) diluted 1:1000 in blocking solution for 60 min at room temperature. F-Actin filaments were counterstained with Alexa Fluor 594 Phalloidin (1:500 Life Technologies A12381) in PBS for 45 min at room temperature. Each slide was then mounted in Vectashield with DAPI (Vector Labs) to counterstain nuclei. For image acquisition, a Nikon C2 Confocal Microscope System equipped with a Plan-Apochromat λ 60X/ 1.40 oil objective was used.

mRNA, microRNA targets analysis and quantitative PCR

Genes of interest were analysed by real time PCR using specific primers, as follows: Rap2c FW 5'-ATGAGGGAATACAAGGTAGTGGT-3' and REV 5'-ACTTCGATCTCTTTGCGGTAGA-3'; Src FW 5'-CAATGCCAAGGGCCTAAATGT-3' and REV 5'-TGTTTGGAGTAGTAAGCCACGA-3'; Itga1 FW 5'-CCTTCCCTCGGATGTGAGTCA-3' and REV 5'-AAGTTCTCCCCGTATGGTAAGA-3'; Col4a4 FW 5'-CGGGCTAACAGCATTTACTGC-3' and REV 5'-CCAGAAAGGGGGACTGGAGT-3'; Col4a5 FW 5'-GAAAAAGGCG AACAAGGTCTTC-3' and REV 5'-CAGGAGGCCCTCTATCACCA-3'; Pik3cb FW CTATGGC AGACAACCTTGACAT-3' and REV 5'-CTTCCCGAGGTACTTCCAACT-3'; Arhgef3 FW 5'-TCAAACCCCTTTCCAGAGTCA-3' and REV 5'-TCTCCGCTTTGTGCTTGAGG-3'; RPL37 FW 5'-CGCAAGAGGAAGTATAACTGGAG-3' and REV 5'-TCTGAATCTGCGGTAGACAATCT-3'. Annealing temperature was set at 60° C for all samples.

Single cell spectroscopy experiments using atomic force microscopy (AFM)

Biomechanical experiments were carried out through a Solver Pro-M AFM from NT-MDT (Moscow Russia), as previously reported(1,2). For these measurements, sQube CP-PNPL-Au-C cantilevers were used, with a nominal spring constant of 0.08 N/m, which was checked prior each experiment by Sader method. A spherical gold probe of about 5 μm-diameter was glued at the cantilever's apex. Measurements on single living cells were performed in physiological conditions of medium and temperature, within one hour. Since the nuclear elasticity is correlated with the stages of cell division(3), cells with nuclei optically showing mitosis were excluded. For each investigated area, a preliminary scan was made to assess the cell morphology and the nuclear position, which corresponds to the highest portion of the cell. In order to avoid possible artefacts due to substrate stiffness and/or due to hydrodynamic forces, indentations were performed above the nucleus, at the constant speed of 1 μm/s for approach and withdrawal of the cantilever.

Cell elasticity was calculated from the first portion of the indentation curve (10% of cell deformation). Here, the experimental data were fitted with the model proposed by Sneddon for spherical probes (4):

$$F = \frac{E}{\left(1\text{-}\upsilon^2\right)} \cdot \left(\frac{R^2 + a^2}{2} \cdot \log \frac{R+a}{R-a} - aR \right) \qquad (1)$$

$$\delta = \frac{a}{2} \cdot \log \frac{R+a}{R-a} \qquad (2)$$

Where F is the loading force, E is the Young's modulus, ν the Poisson's ratio, R the radius of the probe, a is the contact radius (function of the tip penetration) and δ is the probe penetration into the cell. Approximating the cell to an incompressible body (i.e. a flexible object filled with liquid), the Poisson's ratio was assumed as $\nu = 0.5$ (5). All the curves analysis was performed using AtomicJ software (6).

The model used for estimating the Young's modulus value is conventionally used to yield a general idea of cell elasticity (7). Other models could be used for this task. One fairly new and accurate is the "Brush Model" developed by Sokolov et al. (8,9). In the present study, it has been decided to use a model which does not consider the brush since (i) this model is the one most commonly used, (ii) the

cell line was the same throughout the all research and (iii) the same protocol/methodology and the same model has been used for all cells and indentations. Therefore, it has been considered that the calculated Young's modulus values might be compared for all specimens within the presented experiments. Furthermore, resulting Young's modulus data are reasonably similar to others reported by another group for the same cell line (10).

To describe the cell viscoelastic behaviour towards an external applied force, we used a parameter introduced by Klymenko et al. (11) and indicated as “plasticity index” η (even though “plastic” stands for non-recoverable deformation). This was assessed from the hysteresis between the approach and withdrawal curves as:

$$\eta = 1 - (A_2/A_1) \qquad (3)$$

where A_1 and A_2 are the areas under the loading and unloading curves (green box in Figure S1), respectively. Intermediate values between a fully elastic (η=0) and a fully plastic behaviour (η=1), indicate mixed viscoelastic properties.

For both the Young’s modulus and the plasticity index assessment, each cell was subjected to three consecutive indentations at the same position and the mean of the results was considered as a single cell value (n=1).

Cell-to-ECM protein interaction was assessed through a JPK NanoWizard II AFM equipped with a CellHesion module, using tipless V-shaped silicon nitride gold covered cantilevers having nominal spring constant value of 0.32 N/m (NanoWorld, Innovative Technologies). O_2 plasma treated cantilevers were functionalized with fibronectin (Thermo Fisher Scientific) at the final concentration of 20 µg/ml for 15 hrs at 4 °C, and stored in PBS (12). Before each experiment, the cantilever spring constant was calibrated using the thermal noise method. Measurements were performed according to published protocols(13-15) (Figure S2). Briefly, HL-1 cell suspension was overlaid on a BSA coated glass coverslip inserted into a petri dish previously coated with type I collagen or fibronectin (both from Thermo Fisher Scientific) at the final concentration of 50 and 20 µg/ml, respectively. A single

cell from the suspension was captured by pressing it with the fibronectin-functionalized cantilever against the glass for 30 sec with a contact force of 0.5 nN. Next, the cell was lifted up from the surface and allowed establishing a firm adhesion to the cantilever for about 20 minutes. Afterwards, the cantilever with the cell was moved far away reaching a coated plastic surface and the adhesion measurements were performed at a constant force of 0.5 nN for 20 seconds. After each force measurement, the cell was retracted to recover for 60 seconds before repeating the measure on the same spot or adhering to a different spot on the surface. During contact, the force exerted was kept constant using the AFM closed loop feedback mode. The cantilever was withdrawn at constant speed of 5 μm/s over pulling ranges of 80 μm to ensure complete detachment of the cell from substrate. During this step, the cantilever deflection, which is proportional to the vertical force that exists between the cell and substrate, is recorded in a force-distance curve. This curve provides information regarding the cell adhesion. The work that is required to detach the cell can be used to describe the adhesion strength of the cell. It is calculated from the area that is enclosed by the retraction- force–distance curve. This curve also carries other information: after the cell starts to detach from the substrate, individual force steps can be observed. During this phase, the receptors either detaches from the substrate surface or are pulled away from the cell cortex as a membrane tether. While cell membrane is still in contact with the substrate, either of these processes can occur. During the final phase of detachment, the cell body is no longer in contact with the substrate and, thus, attachment is due only to the tethers. Since the receptors are anchored in the cell cortex they unbind as the force increases (in the AFM curve they are denoted as jumps or ruptures). The second type of unbinding event occurs when membrane tethers are pulled out of the cell. In this case, long plateaus of constant force characterize tethers. Our withdrawn (unloading) curves were analyzed classifying receptors detachment events as “rupture” or “tether” based on the slope of the curve preceding the force step. Withdrawn curves were analysed using the JPK Data Processing software. Positive steps at the right of the minimum force were automatically identified (using default fit parameters values: “Smoothing” = 5; “Significance” = 0.001). Detachment events were classified as “rupture” or “tether” based on the

slope of the curve preceding the force step as follows: values ≤ -0.15mN/m, corresponding to the 15% derivation were associated to ruptures, while slopes between -0.15 and 0.15 mN/m were classified as tethers(14). Intervals between steps lower than 12.5 nm were discarded, as fit was made only on two experimental points. For these experiments, each cell was subjected to 4-6 consecutive indentations at the same collagen or fibronectin spot and the mean of the results was considered as a single cell-ECM interaction (n=1).

Actin aggregates

A quantification of the actin aggregates amount has been done using imageJ software. The calculations were performed for every cell line on 20 cells from 4 independent experiment. The results are shown in the figure below and presented as the amount of actin aggregates on the total cytoskeleton area. They are: (median: NT = 1.08%, WT = 0.97%, PKP2 = 2.90%, PKP2/antimiR= 2.59%)(*p* value calculated using unpaired Wilcoxon-Mann-Whitney test (confidence level 95%), NT vs WT: p-value = 0.4652, NT vs PKP2: p-value = 1.466e-07, NT vs antimiR: p-value = 1.466e-07, WT vs PKP2: p-value = 1.475e-07, WT vs antimiR: p-value = 1.475e-07, PKP2 vs antimiR: p-value = 0.04873).

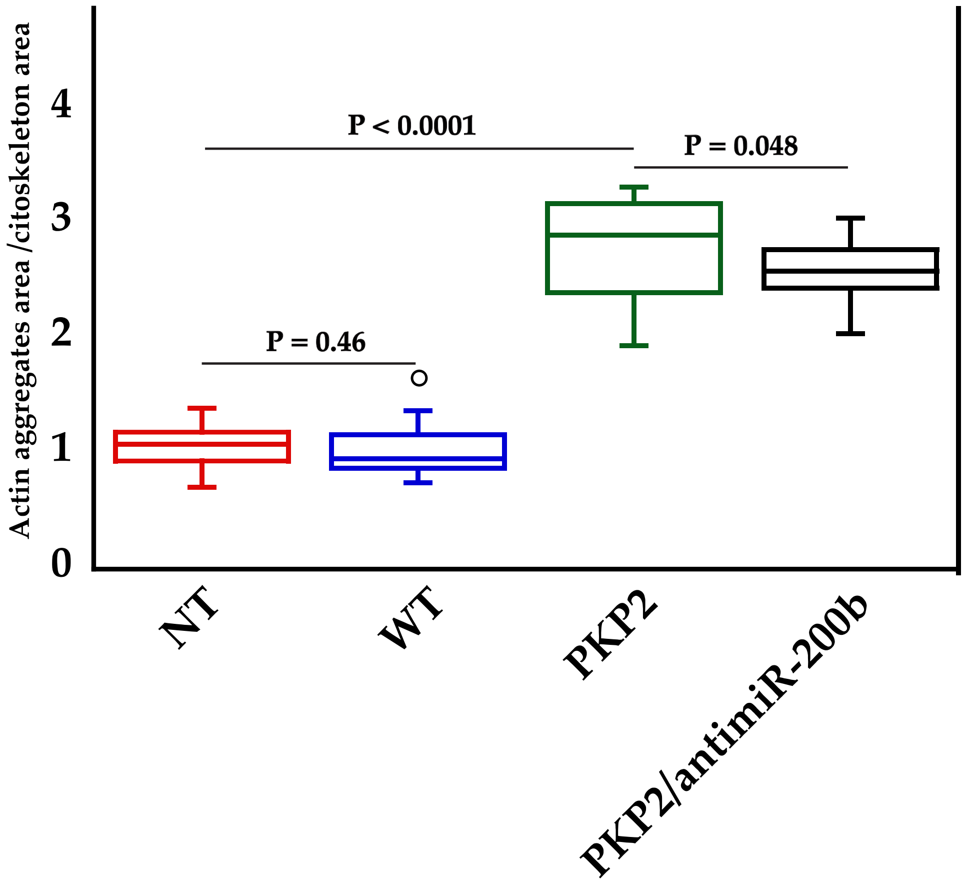

Supplementary Figure S1. A quantification of the actin aggregates amount found in the cells cytoskeleton

SUPPLEMENTARY REFERENCES

1. Puzzi L, Borin D, Martinelli V, Mestroni L, Kelsell DP, Sbaizero O. Cellular biomechanics impairment in keratinocytes is associated with a C-terminal truncated desmoplakin: An atomic force microscopy investigation. Micron. 2018;106:27-33.

2. Borin D, Puzzi L, Martinelli V, Cibinel M, Lapasin R, Sbaizero O. An engineering insight into the relationship of selective cytoskeletal impairment and biomechanics of HeLa cells. Micron. 2017;102:88-96.

3. Matzke R, Jacobson K, Radmacher M. Direct, high-resolution measurement of furrow stiffening during division of adherent cells. Nature cell biology. 2001;3(6):607-10.

4. Sneddon IN. The relation between load and penetration in the axisymmetric boussinesq problem for a punch of arbitrary profile. Int J Eng Sci. 1964;3:47-57.

5. Radmacher M. Measuring the elastic properties of living cells by the atomic force microscope. Methods in cell biology. 2002;68:67-90.

6. Hermanowicz P, Sarna M, Burda K, Gabrys H. AtomicJ: an open source software for analysis of force curves. The Review of scientific instruments. 2014;85(6):063703.

7. Carl P, Schillers H. Elasticity measurement of living cells with an atomic force microscope: data acquisition and processing. Pflugers Archiv : European journal of physiology. 2008;457(2):551-9.

8. Guz N, Dokukin M, Kalaparthi V, Sokolov I. If cell mechanics can be described by elastic modulus: study of different models and probes used in indentation experiments. Biophysical journal. 2014;107(3):564-75.

9. Dokukin M, Ablaeva Y, Kalaparthi V, Seluanov A, Gorbunova V, Sokolov I. Pericellular Brush and Mechanics of Guinea Pig Fibroblast Cells Studied with AFM. Biophysical journal. 2016;111(1):236-46.

10. Kossivas F, Michaelides M, Hadjisavvas A, Kyprianou A, Kyriacou K, Constantinides C. Elasticity Measurements from Left Ventricular Murine HL-1 Cardiomyocytes using Atomic Force Microscopy. The FASEB Journal. 2015;29(1_supplement):799.8.

11. Klymenko OW-Z, J.; Lekka, M; Kwiatek, W.M. Energy Dissipation in the AFM Elasticity Measurements. Acta Physica Polonica A. 2009;115:4.

12. Bharadwaj M, Strohmeyer N, Colo GP, Helenius J, Beerenwinkel N, Schiller HB, et al. alphaV-class integrins exert dual roles on alpha5beta1 integrins to strengthen adhesion to fibronectin. Nature communications. 2017;8:14348.

13. Andolfi L, Bourkoula E, Migliorini E, Palma A, Pucer A, Skrap M, et al. Investigation of adhesion and mechanical properties of human glioma cells by single cell force spectroscopy and atomic force microscopy. PloS one. 2014;9(11):e112582.

14. Chu C, Celik E, Rico F, Moy VT. Elongated membrane tethers, individually anchored by high affinity alpha4beta1/VCAM-1 complexes, are the quantal units of monocyte arrests. PloS one. 2013;8(5):e64187.

15. Friedrichs J, Helenius J, Muller DJ. Quantifying cellular adhesion to extracellular matrix components by single-cell force spectroscopy. Nature protocols. 2010;5(7):1353-61.

Supplementary Table 1. Summary of the genes altered by PKP2 downregulation. Overlap between miR-200 targets genes and dysregulated genes in HL-1^{PKP2} detected in RNA-seq analysis revealed that out of 816 differential expressed potential targets, 560 genes were suppressed ($p<0.001$).

Gene-ID	Gene Symbol	MG33304	MG33303	MG33306	MG33305	q_value	FC
Krt80	Krt80	0.02	0.03	0.93	0.6	0.022243	30.5425
Mgat3	Mgat3	0.02	0	0.39	0.4	1.66E-05	21.89637
4932418E24Rik	4932418E24Rik	0.08	0.04	1.16	1.16	0.040599	17.88034
Nptx1	Nptx1	0.04	0.05	0.51	0.53	6.95E-08	12.47215
RGS3	Rgs3	1.23	0.89	10.78	10.88	0	8.426225
Hivep3	Hivep3	1.3	1.68	2.76	2.54	0.000128	7.672605
Hivep3	Hivep3	0.14	0.06	0.07	0.12	0.000128	7.672605
Zcchc24	Zcchc24	0.81	0.82	6.55	6.17	0	7.519725
TIMP3	Timp3	0.24	0.3	2.14	1.82	0	6.783645
PODXL	Podxl	0.72	0.74	5.21	4.5	0	6.390263
NOTCH1	Notch1	0.2	0.16	1.11	0.9	0	5.037838
THBD	Thbd	2.78	2.3	13.38	12.28	0	4.944279
ETS1	Ets1	0.16	0.09	0.59	0.55	7.29E-08	4.908215
ZCCHC3	Zcchc3	0.17	0.11	0.86	0.57	7.02E-06	4.790255
Hlf	Hlf	0.2	0.11	0.83	0.8	1.27E-08	4.680739
Fstl4	Fstl4	0.05	0.03	0.35	0.29	0.011022	3.371436
E130309F12Rik	E130309F12Rik	0.7	0.87	2.63	2.56	3.47E-10	3.250179
Rps6ka2	Rps6ka2	5.52	5.19	17.25	15.95	0	3.023525
DUSP5	Dusp5	1.56	1.05	4.31	3.6	9.97E-15	2.965387
MAP3K6	Map3k6	0.53	0.42	1.4	1.08	9.38E-07	2.921686
Dact1	Dact1	0.59	0.53	1.78	1.67	0.00013	2.918841

Gene-ID	Gene Symbol	MG33304	MG33303	MG33306	MG33305	q_value	FC
PTPRM	Ptprm	0.27	0.46	0.99	1	8.75E-10	2.879424
PLAU	Plau	0.52	0.59	1.77	1.35	1.17E-05	2.840903
Napb	Napb	1.76	2	5.43	5.2	8.86E-09	2.65058
PHF15	Phf15	0.46	0.37	1.15	0.98	2.57E-05	2.576895
Frem1	Frem1	2.53	2.96	6.88	7.25	0	2.465527
Jag2	Jag2	0.82	0.95	2.44	2.14	3.05E-08	2.458426
Fhl1	Fhl1	23.75	23.13	60.26	58.78	0	2.446972
SLC2A3	Slc2a3	2.58	2.22	5.37	4.82	1.18E-05	2.383514
ACOT7	Acot7	19.57	17.59	47.11	42.26	0	2.336728
NACC2	Nacc2	1.76	1.45	3.9	3.66	1.9E-11	2.313103
ASAP2	Asap2	0.2	0.21	0.48	0.42	0.048604	2.29226
Cacna1c	Cacna1c	3.06	3.32	7.46	6.67	0	2.286936
WNK1	Wnk1	12.74	11.93	30.67	27.05	0	2.274337
Phactr2	Phactr2	10.65	10.91	24.67	24.02	0	2.273698
ZBED4	Zbed4	3.01	2.77	7.39	6.59	0	2.265985
GNB4	Gnb4	1.84	2.05	4.51	4	0.005311	2.200025
Rfx3	Rfx3	1.07	1.06	2.17	2	4.23E-06	2.072599
SLC35F2	Slc35f2	1.59	1.42	3.46	3.18	1.59E-05	2.072078
REEP5	Reep5	19.26	18.47	42.54	39.59	0	2.063445
Taf4a	Taf4a	4.51	4.42	9.49	8.61	2.51E-11	2.034499
Gpr161	Gpr161	0.73	0.68	1.35	1.37	0.000429	2.029302
HIPK2	Hipk2	2.85	2.85	6.16	5.53	1.13E-08	2.014495
LRP11	Lrp11	0.37	0.24	0.64	0.55	0.014825	1.968097
CAP2	Cap2	16.72	15.69	33.18	32.56	0	1.966595
Glcci1	Glcci1	2.77	2.18	5.42	7.48	2.45E-10	1.964746

Gene-ID	Gene Symbol	MG33304	MG33303	MG33306	MG33305	q_value	FC
Nova1	Nova1	0.32	0.4	0.83	0.72	5.06E-05	1.949492
TNFRSF21	Tnfrsf21	1.2	1.06	2.35	2.17	2.24E-05	1.936216
Slc24a4	Slc24a4	1.16	1.1	2.17	2.44	0.003401	1.9115
ELK3	Elk3	0.27	0.48	0.7	0.63	0.029069	1.872517
ADIPOR2	Adipor2	24.6	24.5	48.98	45.78	0	1.871734
INPP1	Inpp1	2.13	1.95	4.35	3.75	0.001038	1.859247
Sbf1	Sbf1	6.34	6.02	12.56	11.25	5.69E-14	1.856658
SLC38A9	Slc38a9	0.59	0.57	1.01	1.01	0.002332	1.854167
Hivep3	Hivep3	1.3	1.68	2.76	2.54	0.03627	1.828735
Hivep3	Hivep3	0.14	0.06	0.07	0.12	0.03627	1.828735
KLHDC10	Klhdc10	29.88	28.64	55.52	55.67	0	1.827731
WDFY1	Wdfy1	4.69	4.36	9.06	8.2	0.007871	1.827057
MAP3K1	Map3k1	2.69	2.06	4.76	3.86	5.22E-07	1.813242
PSMF1	Psmf1	14.38	13.95	30.04	27.98	0	1.797353
Fyn	Fyn	1.13	1.31	2.15	2.02	0.005718	1.777707
DNAJC5	Dnajc5	27.09	25.77	50.23	46.31	9.97E-15	1.753041
PDE4A	Pde4a	3.76	2.66	5.77	5.34	3.67E-05	1.752007
Tln2	Tln2	1.86	1.81	3.26	3.05	2.95E-09	1.735057
TOMM34	Tomm34	30.64	28.94	54.4	52.18	0	1.729104
ARID2	Arid2	2.63	2.67	4.81	5.09	2.24E-11	1.716535
Tmem170	Tmem170	1.5	1.08	1.4	1.26	0.025376	1.713441
MED30	Med30	22.92	25.61	40.67	45.31	1.31E-11	1.710977
MEF2A	Mef2a	21.3	21.73	39.99	36.74	6.75E-13	1.701665
Ncoa7	Ncoa7	0.62	0.85	1.55	1.46	0.010067	1.699557
Zeb1	Zeb1	3.3	3.07	6.37	5	3.34E-06	1.694834

Gene-ID	Gene Symbol	MG33304	MG33303	MG33306	MG33305	q_value	FC
MTMR12	Mtmr12	7.04	6.85	13.21	11.63	1.48E-11	1.689288
NUDCD1	Nudcd1	7.22	7.24	12.53	13.07	3.07E-06	1.668957
HES1	Hes1	5.16	4.58	7.47	8.31	2.68E-05	1.668102
ARHGAP1	Arhgap1	34.08	30.49	57.43	52.22	2.04E-13	1.667268
NECAP2	Necap2	20.53	19.93	37.25	35.1	1.7E-12	1.665595
PTPN1	Ptpn1	9.5	8.63	19.47	16.77	2.48E-12	1.657331
Dennd1b	Dennd1b	8.74	9.19	18.78	18.11	1.13E-11	1.643506
CDKN1B	Cdkn1b	28.94	28.96	49.49	49.65	2.59E-10	1.63941
CAST	Cast	18.66	18.24	31.19	30.64	1.33E-08	1.636178
ELMO2	Elmo2	11.06	10.07	19.07	18.07	1.13E-08	1.6325
LIMS1	Lims1	19.61	20.01	39.75	38	1.91E-13	1.631916
Ell2	Ell2	4.19	4.35	7.2	7.09	3.1E-08	1.630162
HDAC4	Hdac4	2.8	2.91	4.96	4.36	2.44E-06	1.620895
HDAC4	Hdac4	4.3	3.86	7.18	6.06	2.44E-06	1.620895
Sox12	Sox12	3.2	2.92	5.41	4.68	2.35E-07	1.609669
RALGAPB	Ralgapb	9.23	10.74	17.99	15.96	5.02E-11	1.608122
JAZF1	Jazf1	1.63	1.42	2.59	2.49	0.002097	1.60726
ENC1	Enc1	0.99	0.98	1.77	1.57	0.000576	1.603919
Snrpb2	Snrpb2	20.52	24.5	40.42	40.04	1.67E-10	1.591182
ASXL1	Asxl1	9.44	9.29	15.98	14.88	2.4E-10	1.59111
Dgka	Dgka	2.24	2.43	3.57	4.28	0.002759	1.58848
Aim1	Aim1	0.71	0.55	0.89	0.97	0.038293	1.588385
LGR4	Lgr4	5.04	5.71	9	8.77	3.15E-06	1.573989
PARD6B	Pard6b	1.64	1.38	2.35	2.53	0.00077	1.568525
DCP1A	Dcp1a	4.8	4.8	7.92	7.59	5.1E-07	1.557783

Gene-ID	Gene Symbol	MG33304	MG33303	MG33306	MG33305	q_value	FC
PLAGL2	Plagl2	1.46	1.14	2.13	1.72	0.000611	1.557011
ITM2C	Itm2c	46.56	44.6	73.42	70.75	2.13E-12	1.54581
Ipo8	Ipo8	8.7	8.9	14.82	14.43	6.6E-08	1.532839
KLF10	Klf10	10.5	10.72	16.48	17.13	8.98E-09	1.532376
MANBAL	Manbal	12.8	13.13	21.53	21.17	2.68E-06	1.530318
Prdm16	Prdm16	2.29	1.6	2.83	3.13	0.027582	1.525588
FN1	Fn1	3.22	2.94	5.13	4.36	3.62E-05	1.525467
Clasp1	Clasp1	27.48	26.79	42.72	39.72	2.53E-10	1.520238
GPX8	Gpx8	6.71	8.58	12.17	10.05	0.003036	1.515593
ADNP	Adnp	1.56	1.54	2.77	2.2	0.004371	1.514231
MRE11A	Mre11a	12.17	11.3	17.65	15.63	0.000061	1.510015
Rnf5	Rnf5	6.75	6.53	10.46	10.2	0.000705	1.49724
BSG	Bsg	402.35	377.61	611.85	584.55	8.29E-10	1.495822
GCLC	Gclc	8.02	7.92	12.1	12.62	2.56E-05	1.492475
NOL12	Nol12	8.47	8.13	11.34	12.83	0.004173	1.482285
VPS13B	Vps13b	4.29	4.59	7.17	6.51	4.14E-07	1.474251
TSGA14	Tsga14	2.74	2.73	4.74	4.07	0.02658	1.472676
IFRD2	Ifrd2	14.99	13.38	23.3	20.87	6.51E-07	1.472085
AES	Aes	105.3	90.82	151.1	142.66	3.7E-10	1.471443
SMARCAD1	Smarcad1	4.24	4.75	6.56	7.09	0.010732	1.471292
Ppm1b	Ppm1b	23.52	22.2	36.08	34.84	8.83E-06	1.467686
Immp2l	Immp2l	3.64	3.57	5.54	6.57	0.049354	1.467317
SERPINB9	Serpinb9	2.49	2.05	3.37	3.58	0.00372	1.466848
RAB22A	Rab22a	11.32	10.16	15.55	15.01	1.03E-05	1.466622
Pbx3	Pbx3	12.37	12.9	19.39	17.86	0.000271	1.456454

Gene-ID	Gene Symbol	MG33304	MG33303	MG33306	MG33305	q_value	FC
POFUT1	Pofut1	7.76	7.49	12.29	10.94	3.34E-06	1.454448
HYLS1	Hyls1	7.41	8.69	11.76	12.34	0.007821	1.447862
TMEM65	Tmem65	9.25	8.97	14.11	13.8	4.74E-06	1.43896
Pkia	Pkia	19.44	21.07	30.5	31.38	2.31E-08	1.435706
SCRN1	Scrn1	1.81	1.79	2.75	2.53	0.038541	1.431997
Jmy	Jmy	3.92	3.82	6.21	5.4	0.000379	1.43144
GOLT1B	Golt1b	19.02	20.46	30.29	29.47	1.95E-07	1.42988
Fam57a	Fam57a	8.96	8.23	13.11	11.69	0.034183	1.419681
Six1	Six1	2.32	1.39	2.94	2.21	0.005392	1.418853
ADAR	Adar	6.19	5.98	8.59	9.6	0.0006	1.418582
Zfp217	Zfp217	2.65	2.39	3.96	3.38	0.014816	1.417933
YTHDF1	Ythdf1	15.48	16.82	24.52	23.09	3.03E-07	1.415641
QSOX2	Qsox2	5.8	5.66	8.85	8.01	0.00042	1.415608
SPATA2	Spata2	5.51	5.53	8.47	7.9	0.033395	1.414062
Chd2	Chd2	7.35	7.77	11.04	10.08	8.66E-05	1.413159
St13	St13	65.58	71.61	101.68	98.81	7.27E-06	1.412346
Ppp2r3a	Ppp2r3a	7.86	8.31	11.27	12.09	0.000106	1.411153
DNA2	Dna2	8.41	9.07	12.79	12.9	7.77E-06	1.410956
SLC25A30	Slc25a30	10.22	10.58	15.57	14.29	0.000736	1.409991
Rps6ka6	Rps6ka6	5.82	5.64	8.43	7.16	0.00107	1.40845
DDAH1	Ddah1	0.88	0.94	1.85	1.36	0.047534	1.407905
Kras	Kras	5.78	7.17	10.7	9.83	0.000837	1.406761
MTDH	Mtdh	31.15	34.5	47.3	50.05	1.17E-06	1.4043
Bcl2	Bcl2	1.61	1.5	2.39	1.99	0.025099	1.400664
CBFA2T2	Cbfa2t2	6.18	5.93	9.69	9.55	0.001042	1.398412

Gene-ID	Gene Symbol	MG33304	MG33303	MG33306	MG33305	q_value	FC
AMPD3	Ampd3	9.89	8.85	14.07	12.22	0.001873	1.397122
NCOA3	Ncoa3	8.55	7.88	12.61	11.05	0.002881	1.39062
CDYL	Cdyl	4.71	4.24	6.54	5.97	0.001061	1.390548
Zfr	Zfr	65.34	68.76	98.31	91.72	1.88E-07	1.387079
Siah1a	Siah1a	5.26	6.24	8.7	8.56	0.00155	1.38564
RERE	Rere	7.78	7.97	12.22	9.44	0.018576	1.383272
GHITM	Ghitm	59.22	62.79	87.11	88.89	1.59E-07	1.383206
ACVR2A	Acvr2a	3.86	3.95	5.39	5	0.006433	1.382212
Mapk7	Mapk7	3.89	3.48	5.52	4.89	0.03634	1.382098
Trp53inp2	Trp53inp2	62.64	64.08	95.33	86.05	4.71E-06	1.380824
TNRC6B	Tnrc6b	3.5	3.68	5.52	4.83	1.13E-05	1.380356
TBC1D4	Tbc1d4	10.91	11.63	17.3	15.2	8.37E-06	1.379721
Lpl	Lpl	104.29	110.98	154.14	145.88	6.93E-07	1.379716
EIF2S2	Eif2s2	78.72	88.59	128.05	120.98	4.23E-07	1.373171
GATA6	Gata6	11.47	11.2	16.4	15.9	0.000611	1.372337
HEXIM1	Hexim1	3.36	3.29	5.28	4.25	0.002684	1.371303
PDRG1	Pdrg1	45.8	44.73	68.42	63.68	0.000013	1.369745
ROCK2	Rock2	15.72	18.25	24.94	23.66	8.27E-07	1.369699
Rad21	Rad21	23.24	23.19	35.13	32.55	1.79E-06	1.368987
Rbm26	Rbm26	7.12	7.89	10.97	10.3	0.00458	1.36703
LPCAT1	Lpcat1	4.85	4.63	7.28	5.58	0.016958	1.366197
PLEKHB2	Plekhb2	18.16	17.33	26.45	24.7	5.83E-06	1.36556
Qk	Qk	35.99	35.37	52.66	48.51	9.26E-05	1.364261
Qk	Qk	0.8	0.65	1.7	1.21	9.26E-05	1.364261
PIP4K2A	Pip4k2a	3.42	2.99	4.39	4.14	0.005905	1.360864

Gene-ID	Gene Symbol	MG33304	MG33303	MG33306	MG33305	q_value	FC
YWHAB	Ywhab	45.47	44.79	68.06	61.49	9.15E-07	1.360272
NFYA	Nfya	10.22	11.26	16.34	14.52	0.007204	1.358941
PDGFA	Pdgfa	13.82	12.83	20.18	19.61	0.014361	1.358684
LPIN2	Lpin2	5.5	4.68	6.86	7.13	0.002166	1.356379
PDLIM1	Pdlim1	23.1	20.77	37.4	29.95	0.000273	1.352694
Sik1	Sik1	9.67	8.57	13.18	12.42	0.000129	1.346371
DERL1	Derl1	29.91	30.5	42.73	42.69	1.23E-05	1.344818
PIP5K1B	Pip5k1b	5.06	5.03	6.87	6.72	0.006247	1.343383
CRLF3	Crlf3	11.91	12.39	17.74	15.95	0.000995	1.339905
Trim44	Trim44	9.67	10.11	14.5	13.69	3.11E-05	1.334779
Ccdc47	Ccdc47	26.54	31.85	38.88	38.3	5.87E-05	1.334071
HNRNPF	Hnrnpf	212	230.91	308.47	306.93	2.27E-05	1.333284
OSBPL8	Osbpl8	4.39	4.93	6.72	6.49	0.000895	1.330613
DLAT	Dlat	43.59	45.34	61.99	57.17	1.33E-05	1.329959
COMMD7	Commd7	23.61	22.89	33.37	31.91	0.002596	1.328677
CDC42EP1	Cdc42ep1	4.46	3.89	6.02	5.33	0.033298	1.325599
EIF5	Eif5	35.43	36.68	52.49	51.06	1.29E-05	1.325425
RASA1	Rasa1	7.03	8.6	10.69	10.63	0.001257	1.324873
ANKRD28	Ankrd28	5.11	4.94	6.93	7.17	0.008186	1.322578
Prkar1a	Prkar1a	59.17	60.32	85.92	82.17	3.89E-05	1.320025
MAPRE1	Mapre1	49.24	50	69.91	74.11	1.19E-05	1.317138
CPEB4	Cpeb4	6.18	6.85	9.11	8.54	0.017815	1.314845
PPP1R12A	Ppp1r12a	38.09	41.28	51.44	52.16	0.000327	1.309776
Secisbp2l	Secisbp2l	3.9	4.15	5.91	5.25	0.001426	1.307054
RFK	Rfk	10.57	11.93	15.08	14.94	0.005091	1.300948

Gene-ID	Gene Symbol	MG33304	MG33303	MG33306	MG33305	q_value	FC
Rfx7	Rfx7	3.08	3.31	4.73	4.21	0.025563	1.293671
BCL2L2	Bcl2l2	6.27	6.89	8.95	9.03	0.00879	1.287121
Prkar2a	Prkar2a	6.91	7.16	10.35	8.77	0.003928	1.285457
LPGAT1	Lpgat1	14.23	15.95	22.77	20.58	0.000206	1.285206
COL4A1	Col4a1	31.34	28.2	41.85	36.55	0.000111	1.280141
NHLRC2	Nhlrc2	10.3	10.2	14.11	13.25	0.012976	1.276292
PUM2	Pum2	54.35	59.79	78.34	76.67	0.000614	1.275763
Tex2	Tex2	15.51	14.81	20.2	18.81	0.000712	1.275253
UBE2E3	Ube2e3	21.86	21.74	28.49	26.52	0.014353	1.274552
ESPL1	Espl1	7.99	7.2	10.82	9.29	0.002664	1.273741
Trio	Trio	3.44	3.41	4.73	4.31	0.016401	1.272414
Ppp2r5c	Ppp2r5c	21.97	22.48	32.08	29.85	0.005096	1.271995
JUND	Jund	10.13	8.94	12.2	12.51	0.003693	1.271845
MBOAT7	Mboat7	11.57	11.84	15.91	13.61	0.005543	1.268972
PRPF40A	Prpf40a	36.32	37.8	47.78	46.55	0.001665	1.265245
HDGFRP3	Hdgfrp3	10.13	10.99	14.23	13.84	0.001959	1.264476
CDC25B	Cdc25b	10.08	9.72	14.22	11.96	0.023384	1.263837
Acyp1	Acyp1	18.47	19.92	25.79	25.62	0.037419	1.261974
MIB1	Mib1	19.21	20.33	27.54	25.73	0.005824	1.25864
NEDD4	Nedd4	116.39	115.31	157.43	147.04	0.001558	1.256904
RIT1	Rit1	26.28	24.01	33.15	31.78	0.015084	1.255991
ARL6IP1	Arl6ip1	94.27	97.09	126.62	126.99	0.00045	1.254665
ZCCHC2	Zcchc2	4.9	4.99	6.71	5.94	0.040483	1.249729
DDX24	Ddx24	26.17	25.48	34.41	32.29	0.001739	1.2492
Fam3c	Fam3c	18.72	17.84	24.15	24.31	0.005624	1.247964

Gene-ID	Gene Symbol	MG33304	MG33303	MG33306	MG33305	q_value	FC
SESN2	Sesn2	7.34	6.17	8.6	8.2	0.035519	1.247867
VPS13C	Vps13c	5.33	5.7	7.31	7.03	0.00454	1.247833
WAPAL	Wapal	18.87	23.27	27.71	26.98	0.004368	1.247131
RMND5A	Rmnd5a	16.04	17.68	24.03	21.69	0.002566	1.246106
BLCAP	Blcap	14.99	15.94	19.95	19.9	0.031309	1.244427
YAP1	Yap1	17.7	17.22	24.05	23.96	0.007612	1.24396
Nbr1	Nbr1	24.63	26.27	32.39	32.38	0.002116	1.243403
DENND5B	Dennd5b	5.42	5.55	8.18	7.4	0.009152	1.240311
CTSC	Ctsc	13.89	13.73	17.27	15.16	0.016239	1.239215
Aplp2	Aplp2	57.89	54.05	75.8	70.11	0.002876	1.230864
HMOX1	Hmox1	18.84	17.28	24.2	20.99	0.016205	1.228884
VEGFA	Vegfa	20.37	19.55	25.21	25.39	0.039137	1.227264
USP4	Usp4	17.95	16.76	22.75	21.62	0.007121	1.22262
Tbc1d2b	Tbc1d2b	5.3	4.94	7.09	6.06	0.018093	1.22252
Myo9a	Myo9a	5.81	5.96	7.49	7.49	0.021506	1.219239
Fam60a	Fam60a	12.72	13.43	16.36	16.8	0.021772	1.217956
PHIP	Phip	7.43	8.09	10.29	9.57	0.01139	1.213597
RND3	Rnd3	26.26	27.12	33.79	34.36	0.0083	1.206129
KIF13A	Kif13a	9.04	8.58	11.55	10.37	0.041871	1.205663
Fxr2	Fxr2	30.95	29.11	37.72	37.49	0.007404	1.205155
ZFP106	Zfp106	16.28	14.65	20.89	20.06	0.015302	1.203638
CALM1	Calm1	115.87	98.23	152.94	135.14	0.010239	1.2011
PPP1R11	Ppp1r11	45.42	41.22	58.65	49.63	0.026819	1.200068
ATP5G3	Atp5g3	464.59	446.23	578.86	573.04	0.008199	1.20004
KBTBD2	Kbtbd2	15.22	15.57	19.61	19.04	0.034227	1.196404

Gene-ID	Gene Symbol	MG33304	MG33303	MG33306	MG33305	q_value	FC
IDI1	Idi1	27.35	29.35	38.5	34.65	0.011444	1.193936
Zc3hav1	Zc3hav1	25.01	25.96	31.58	30.75	0.040241	1.191238
PRNP	Prnp	32.57	31.06	39.94	38	0.018343	1.187896
Sdc2	Sdc2	19.39	20.33	25.27	23.77	0.019589	1.187068
Slc38a2	Slc38a2	21.71	21.96	28.81	26.14	0.02516	1.187054
BC005537	BC005537	13.49	14.42	18.68	16.57	0.046808	1.183772
MRPS35	Mrps35	39.47	41.61	51.07	49.02	0.041739	1.179813
OAZ1	Oaz1	351.07	314.32	414.43	401.67	0.017999	1.176567
IPO5	Ipo5	96.46	98.05	123.03	115.06	0.047865	1.155842
FKBP9	Fkbp9	76.91	74.13	71.4	66.82	0.043604	-1.15186
ADAM19	Adam19	16.92	16.17	15.56	13.52	0.038945	-1.16135
Ptbp2	Ptbp2	33.13	35.71	32.21	30.56	0.045393	-1.16263
DYNC1LI2	Dync1li2	44.92	47.43	42.83	40.69	0.023627	-1.16876
PCNP	Pcnp	47.12	52.41	46.89	46.81	0.033753	-1.16951
Prmt7	Prmt7	66.04	60.75	57.15	54.45	0.020486	-1.17796
FBXW11	Fbxw11	19.59	18.38	18.47	16.66	0.049596	-1.17809
PTP4A2	Ptp4a2	112.67	119.22	99.86	103.98	0.018603	-1.17874
PJA2	Pja2	17.99	18.34	16.7	15.76	0.036692	-1.17924
Eri1	Eri1	15.63	16.6	14.3	14.24	0.023167	-1.17937
TAX1BP1	Tax1bp1	66.47	72.15	61.03	61.05	0.01324	-1.1837
Crk	Crk	27.52	27.63	25.34	23.81	0.021635	-1.18719
DNAJC13	Dnajc13	9.28	10.05	8.89	8.25	0.023656	-1.18732
Arhgef17	Arhgef17	15.32	13.07	13.37	11.25	0.012101	-1.18782
ATP11B	Atp11b	8.32	9.12	7.76	7.51	0.040945	-1.18835
CFL2	Cfl2	84.78	89.63	80.13	77.24	0.012023	-1.1897

Gene-ID	Gene Symbol	MG33304	MG33303	MG33306	MG33305	q_value	FC
MED13	Med13	7.01	7.17	6.57	6.4	0.019528	-1.19173
BUB3	Bub3	92.34	83.43	80.66	74.83	0.009184	-1.19313
Pten	Pten	30.55	33.17	28.71	28.24	0.008697	-1.19404
SDPR	Sdpr	97.22	92.04	83.53	80.33	0.008601	-1.19595
NAE1	Nae1	58.76	68.65	56.5	56.22	0.009588	-1.1979
LARP4B	Larp4b	16.48	17.86	15.48	14.88	0.014993	-1.199
CHSY1	Chsy1	7.84	7.87	7.23	6.53	0.030751	-1.2001
IQGAP1	Iqgap1	46.98	44.31	40.92	37.64	0.007753	-1.20057
HSPA5	Hspa5	482.06	488.1	427.57	416.02	0.036181	-1.20164
SFPQ	Sfpq	84.24	85	74.57	72.39	0.032389	-1.20272
PARP1	Parp1	66.29	66.9	59.67	54.01	0.008709	-1.20301
NHP2L1	Nhp2l1	120.53	117.67	107.69	100.84	0.00527	-1.20779
UHRF1BP1	Uhrf1bp1	5.66	5.87	5.46	4.54	0.015242	-1.21087
FAM178A	Fam178a	13.99	15.06	12.61	12.29	0.009797	-1.21356
MAZ	Maz	42.28	36.84	35.36	30.75	0.011051	-1.21515
H6PD	H6pd	5.71	5.3	4.82	4.58	0.036328	-1.21524
KLF6	Klf6	5.32	5.15	4.71	4.35	0.042641	-1.21686
Rap1b	Rap1b	16.38	16.51	15.32	13.4	0.023905	-1.2175
Gdi2	Gdi2	80.7	84.19	73.59	68.64	0.012761	-1.21806
Pip4k2b	Pip4k2b	5.06	4.5	4.24	3.6	0.041651	-1.21915
CRKL	Crkl	12.98	12.52	11.45	10.14	0.013131	-1.21975
DPY19L1	Dpy19l1	17.44	17.02	15.11	14.64	0.026387	-1.2201
CLSPN	Clspn	15.62	16.39	14.09	13.65	0.020316	-1.22108
GNG12	Gng12	18.56	18.1	16.26	17.81	0.032172	-1.22286
MCL1	Mcl1	36.64	37.45	33.59	32.19	0.002867	-1.22364

Gene-ID	Gene Symbol	MG33304	MG33303	MG33306	MG33305	q_value	FC
HDLBP	Hdlbp	93.56	92.57	80.85	76.74	0.007967	-1.22406
EZR	Ezr	83.58	80.77	70.67	63.65	0.004895	-1.22412
IGF1R	Igf1r	9.22	8.12	8.22	7.27	0.016178	-1.22473
SLBP	Slbp	48.32	50.46	44.56	45.76	0.005125	-1.22502
Zranb2	Zranb2	44.83	50.84	40.98	41.95	0.008972	-1.22722
UBXN4	Ubxn4	42.48	45.29	37.73	35.85	0.002153	-1.22725
Cnot8	Cnot8	29.26	29.99	26.42	24.93	0.008049	-1.22905
AFF4	Aff4	23.25	26.8	21.93	20.18	0.005788	-1.22908
Pin1	Pin1	33.92	31.73	27.92	26.66	0.002129	-1.23083
LEPROTL1	Leprotl1	12.24	12.19	9.99	10.09	0.011898	-1.2327
TMEM30A	Tmem30a	26.88	28.74	26.69	24.98	0.002509	-1.23321
DCK	Dck	10.92	11.43	9.44	9.77	0.013327	-1.23354
SLC7A6	Slc7a6	22.84	21.66	20.31	17.29	0.021428	-1.23428
GORASP2	Gorasp2	29.61	27.84	25.25	22.82	0.009766	-1.2346
RABEP1	Rabep1	24.07	27.23	21.64	20.61	0.035058	-1.23561
Ddx1	Ddx1	167.8	170.72	147.37	141.35	0.001933	-1.23672
CCDC6	Ccdc6	11.13	10.16	9.13	8.8	0.018619	-1.23789
Tra2b	Tra2b	82.91	80.21	71.11	69.08	0.001221	-1.23886
Rnf11	Rnf11	43.21	41.96	37.42	34.94	0.009038	-1.24006
CD164	Cd164	22.8	23.92	20.42	19.81	0.004302	-1.24027
1810013L24Rik	1810013L24Rik	10.49	10.65	9.4	8.68	0.041342	-1.24082
FAM126A	Fam126a	17.05	17.33	15.68	13.71	0.005748	-1.24085
FRMD6	Frmd6	6.52	6.05	5.22	5.42	0.035613	-1.24105
C78339	C78339	20.08	20.41	16.09	16.24	0.020869	-1.24293
PCBP1	Pcbp1	32.52	28.35	26.32	23.8	0.005304	-1.24674

Gene-ID	Gene Symbol	MG33304	MG33303	MG33306	MG33305	q_value	FC
CHRAC1	Chrac1	58.37	59.22	48.57	48.07	0.004319	-1.24774
Dnajb9	Dnajb9	7.92	7.44	6.86	6.17	0.045802	-1.24991
MCFD2	Mcfd2	64.98	63.9	58.6	52.65	0.00185	-1.25029
HEXA	Hexa	62.36	66.45	52.79	51.98	0.001072	-1.25144
PUM1	Pum1	14.41	12.99	11.53	10.05	0.017499	-1.2518
UBE2I	Ube2i	234.87	230.07	210.6	201.98	0.006719	-1.2519
Bptf	Bptf	8.1	8.8	6.97	6.22	0.00887	-1.25253
Sephs1	Sephs1	15.79	15.68	13.84	13.18	0.012081	-1.25473
LSM14A	Lsm14a	17.55	17.95	16.56	14.67	0.008396	-1.25626
PANK3	Pank3	14.5	15.95	12.99	12.77	0.000656	-1.25743
STAM2	Stam2	9.81	9.32	9	7.29	0.007842	-1.25744
NR2C2	Nr2c2	11.81	12.51	9.44	10.11	0.019591	-1.25801
BNIP3L	Bnip3l	37.81	40.23	45.13	37.45	0.001069	-1.25843
FAM168A	Fam168a	5.53	5.06	4.54	4.1	0.019162	-1.25942
IRAK2	Irak2	7.75	7.66	6.63	6.36	0.032575	-1.26018
GIGYF2	Gigyf2	15.86	17.02	13.85	13.05	0.007765	-1.26233
ORMDL3	Ormdl3	12.77	12.84	11.14	10.16	0.008827	-1.26448
Abcb7	Abcb7	14.24	15.19	12.18	12.12	0.000717	-1.26484
SEC23A	Sec23a	25.61	26.39	22.2	21.31	0.001708	-1.26503
DENND5A	Dennd5a	51.96	51.56	42.8	41.95	0.000307	-1.26651
Otud4	Otud4	30.82	31.37	27.41	26.13	0.000352	-1.26686
Rnf169	Rnf169	7.03	7.29	5.85	5.84	0.01381	-1.26787
PIK3R1	Pik3r1	7.03	7.23	6.07	5.79	0.002776	-1.26883
CCNG2	Ccng2	11.57	11.68	9.57	9.42	0.006407	-1.27001
PRKD3	Prkd3	34.82	37.22	29.76	30.27	0.000519	-1.27007

Gene-ID	Gene Symbol	MG33304	MG33303	MG33306	MG33305	q_value	FC
SNIP1	Snip1	15.17	14.67	13.15	11.52	0.004525	-1.27143
TEAD1	Tead1	15.11	15.59	14.32	12.93	0.006315	-1.27281
CCNL1	Ccnl1	22.39	24.8	21.21	19.54	0.023094	-1.27307
VEZF1	Vezf1	9.4	8.84	7.88	7.5	0.013131	-1.27446
REST	Rest	8.6	9.02	7.43	7.04	0.004246	-1.27469
RHEB	Rheb	47.75	54.89	43.39	43.91	0.001694	-1.27567
IFNGR2	Ifngr2	70.93	68.64	56.33	57.07	0.000165	-1.27647
Rhot1	Rhot1	21.79	23.49	18.95	19.34	0.00354	-1.27904
Ptpn11	Ptpn11	23.63	23.77	20.27	18.12	0.002558	-1.27951
Rps6kb1	Rps6kb1	11.72	13.28	9.83	11.29	0.030984	-1.2797
ZFAND5	Zfand5	31.03	30.6	27.11	24.39	0.00091	-1.28161
SRPK2	Srpk2	9.85	10.51	8.68	8.14	0.002314	-1.28243
Ppp4r2	Ppp4r2	24.13	26.53	21.42	20.35	0.000955	-1.28316
PRKAG2	Prkag2	33.77	29.7	25.26	25.89	0.008025	-1.28402
SMC4	Smc4	86.7	101.67	76.57	76.89	0.000193	-1.28436
Spast	Spast	10.85	11.6	9.73	8.91	0.006495	-1.28442
Ulk2	Ulk2	11.98	12.5	10.24	9.6	0.003053	-1.28589
Nudt4	Nudt4	10.09	10.47	9.04	8.03	0.01381	-1.28596
MDFIC	Mdfic	18.68	19.06	15.64	14.91	0.005806	-1.28756
PPP1CC	Ppp1cc	210.75	215.91	191.39	187.56	0.000108	-1.28772
Piga	Piga	5.8	5.78	5.21	4.51	0.009989	-1.28787
ATP13A3	Atp13a3	19.19	19.1	16.43	15.42	0.00119	-1.28832
Asap1	Asap1	11.76	11.1	9.84	8.28	0.006313	-1.2891
Mybl1	Mybl1	18.6	21.05	15.27	15.44	0.004047	-1.29031
ADD1	Add1	36.79	34.99	30.59	28.65	0.000555	-1.29097

Gene-ID	Gene Symbol	MG33304	MG33303	MG33306	MG33305	q_value	FC
ILDR2	Ildr2	11.6	10.33	9.11	8.66	0.000603	-1.29178
DNMBP	Dnmbp	2.42	2.05	1.75	1.74	0.024789	-1.29296
Fez2	Fez2	15.98	14.95	12.44	12.81	0.002775	-1.29482
Pmpcb	Pmpcb	104.01	105.43	87.64	82.84	6.27E-05	-1.29518
ARHGAP5	Arhgap5	28.5	32.72	25.43	24.6	0.000378	-1.29523
TTF2	Ttf2	14.86	14.47	12.09	11.2	0.000284	-1.29787
TCF12	Tcf12	10.46	10.26	8.59	7.86	0.001732	-1.29918
YTHDC1	Ythdc1	15.74	16.84	13.7	13.21	0.008044	-1.2998
KPNA1	Kpna1	20.27	20.95	16.95	17.14	0.001323	-1.29981
Mpp5	Mpp5	5.48	6.3	4.93	4.65	0.0045	-1.29998
Osbpl11	Osbpl11	9.3	9.42	7.54	7.56	0.002175	-1.30205
TTC3	Ttc3	51.2	57.56	45.78	43.33	0.000346	-1.30244
Kif26b	Kif26b	12.96	12.17	10.27	9.14	0.001464	-1.30338
Slc6a6	Slc6a6	17.62	15.96	14.31	12.41	7.91E-05	-1.3051
MIER1	Mier1	16.27	17.52	13.07	12.78	0.006283	-1.30519
Rab8b	Rab8b	6.95	6.78	5.78	5.33	0.00119	-1.30853
GATAD2B	Gatad2b	6.55	5.49	5.15	4.39	0.002959	-1.30893
CAPN2	Capn2	41.49	40	33.47	30.68	3.67E-05	-1.30955
Ythdf2	Ythdf2	34.07	35.17	28.14	27.21	0.000108	-1.31019
SNX27	Snx27	10.46	9.05	8.22	7.09	0.003575	-1.31023
SPRED1	Spred1	8.8	8.08	7.66	7.3	0.007223	-1.31112
STK17B	Stk17b	14.52	14.99	11.86	11.77	0.000299	-1.31349
ACBD3	Acbd3	16.56	16.76	13.83	12.76	0.000422	-1.31444
BET1L	Bet1l	12.11	11.67	8.98	9.71	0.013182	-1.31581
AGFG1	Agfg1	29.83	29.48	24.47	23.1	0.000837	-1.31604

Gene-ID	Gene Symbol	MG33304	MG33303	MG33306	MG33305	q_value	FC
ATPAF1	Atpaf1	12.58	13.11	11.36	10.64	0.012786	-1.3172
FOSL2	Fosl2	4.75	4.43	3.81	3.34	0.001557	-1.32153
S100pbp	S100pbp	11.95	12.12	10.63	9.98	0.018762	-1.322
FAM160B1	Fam160b1	6.61	6.27	5.36	4.5	0.0066	-1.32254
Ubxn7	Ubxn7	7.28	7.91	6.37	5.77	0.000156	-1.32268
Rnf38	Rnf38	10.17	10	7.26	7.02	0.011698	-1.32314
SACS	Sacs	5.97	6.61	5.07	5.09	0.000621	-1.32329
MESDC1	Mesdc1	7.04	6.71	5.47	5.24	0.001407	-1.32579
PTPN12	Ptpn12	4.74	4.84	4.29	3.83	0.01843	-1.32949
Agap1	Agap1	6.51	5.95	5.21	4.59	0.005685	-1.33005
Ankrd44	Ankrd44	3.82	4.14	3.19	2.96	0.033318	-1.33032
LARP1	Larp1	20.86	18.82	15.79	15.03	8.06E-05	-1.33136
IPO7	Ipo7	93.44	100.37	78.02	75.82	0.000251	-1.33203
Dock9	Dock9	2.6	2.78	2.13	2	0.036976	-1.33205
BNIP3	Bnip3	54.62	53.1	42.63	41.92	0.000121	-1.33259
Yipf5	Yipf5	15.96	15.25	12.79	12.19	0.000331	-1.33544
NOTCH2	Notch2	7.62	6.71	5.99	5.17	2.87E-05	-1.33596
FOXJ3	Foxj3	7.67	7.16	6.77	5.38	0.004748	-1.34301
MAP3K7	Map3k7	20.61	20.63	13.67	14.33	4.32E-05	-1.34394
ABHD10	Abhd10	8.35	9.7	7.36	6.71	0.028375	-1.34543
LGALS8	Lgals8	14.13	14.1	10.58	10.36	0.005441	-1.34622
Lrig1	Lrig1	6.5	6.59	5.77	5.25	0.003309	-1.34674
Rprd1a	Rprd1a	14.96	15.68	12.51	11.42	0.000889	-1.34844
LRRC40	Lrrc40	23.37	21.34	18.8	16.42	0.000052	-1.34971
Ddx3x	Ddx3x	134.19	149.95	115.28	110.37	1.57E-05	-1.35122

Gene-ID	Gene Symbol	MG33304	MG33303	MG33306	MG33305	q_value	FC
Pdik1l	Pdik1l	11.86	12.45	9.69	9.12	0.001611	-1.35208
FRS2	Frs2	3.4	3.78	2.82	2.71	0.008208	-1.35241
Mknk1	Mknk1	16.92	16.78	14.81	12.05	0.006823	-1.35549
AP1S3	Ap1s3	10.16	9.93	7.68	7.31	0.003654	-1.35625
ARL6IP6	Arl6ip6	12.98	14.27	10.66	9.01	0.002093	-1.35652
PKD2	Pkd2	7.26	7.2	5.93	5.52	0.000247	-1.35784
CTDSP2	Ctdsp2	15.92	14.63	12.04	11.24	0.000041	-1.35799
Hsph1	Hsph1	21.27	20.7	16.24	15.4	1.55E-05	-1.35803
UBE2K	Ube2k	29.73	29.48	23.14	23.68	5.35E-05	-1.35808
Pkd1	Pkd1	5.43	4.76	4.11	3.53	0.0001	-1.35901
Lrrc8a	Lrrc8a	9.82	8.8	7.12	6.8	0.00035	-1.35994
Hook3	Hook3	8.05	8.2	5.97	6.17	8.13E-05	-1.36022
Senp5	Senp5	9.34	9.6	7.9	6.89	0.000336	-1.36182
Stard13	Stard13	12.49	11.64	9.33	8.5	6.13E-05	-1.36513
Mboat2	Mboat2	10.36	11.19	8.58	7.99	0.004173	-1.36561
MFHAS1	Mfhas1	11.24	10.43	8.49	7.67	5.71E-05	-1.36585
BAG2	Bag2	37.43	36.87	29.81	26.85	7.4E-06	-1.36905
SEL1L	Sel1l	16.76	16.98	15.36	14.23	0.00013	-1.36946
Cdh20	Cdh20	4.82	4.88	3.72	3.63	0.003347	-1.37015
CTTN	Cttn	55.13	54.66	44.5	41.76	6.84E-07	-1.37044
SIKE1	Sike1	13.81	17.15	12.57	11.24	0.00093	-1.37075
Mtfr1	Mtfr1	22.48	21.45	16.08	16.88	0.000608	-1.3712
USP25	Usp25	24.04	25.66	19.38	18.17	9.27E-06	-1.37136
BHLHE40	Bhlhe40	24.42	23.16	18.63	16.71	4.85E-06	-1.37231
CDC42SE1	Cdc42se1	20.52	19.76	14.46	15.56	0.016672	-1.37278

Gene-ID	Gene Symbol	MG33304	MG33303	MG33306	MG33305	q_value	FC
LMO4	Lmo4	11.19	10.9	8.71	8	0.008267	-1.37314
CHMP5	Chmp5	38.23	41.35	29.82	30.99	2.86E-05	-1.37583
SETX	Setx	16.76	19.19	15.47	12.66	7.61E-06	-1.37631
Sec24a	Sec24a	9.01	8.89	9.62	8.08	0.017896	-1.37688
Hmgb3	Hmgb3	24.23	28.09	21.22	19.72	3.02E-05	-1.37872
FAT1	Fat1	19.1	18.62	15.09	12.99	4.09E-06	-1.37994
TMEM185B	Tmem185b	24.92	24.02	19.47	18.25	6.14E-06	-1.38012
Fmr1	Fmr1	27.56	29.97	23.1	22.75	2.15E-05	-1.38076
EDEM1	Edem1	17.52	17.85	13.86	13.37	3.2E-06	-1.38106
Btf3l4	Btf3l4	49.83	58.97	47.83	44.27	1.05E-05	-1.38345
GSK3B	Gsk3b	12.04	13.11	10.43	8.74	2.37E-06	-1.38375
SMAD7	Smad7	2.74	2.51	2.3	1.87	0.008095	-1.38487
Baz2b	Baz2b	10.48	10.23	7.93	9.8	0.005273	-1.38514
TRIM33	Trim33	5.4	5.85	4.38	4.29	7.46E-05	-1.38527
MAP4K4	Map4k4	32.27	33.3	25.35	23.82	1.77E-06	-1.38735
Bbx	Bbx	19.96	22.45	16.38	15.18	5.47E-06	-1.38753
Lphn2	Lphn2	9.55	9.59	6.9	6.63	0.000446	-1.39042
FNDC3B	Fndc3b	8.39	7.61	6.47	5.52	0.000061	-1.3929
USP32	Usp32	7.46	8.15	6.01	5.79	3.52E-05	-1.3944
Tmem170b	Tmem170b	2.84	3.16	2.3	2.21	0.000987	-1.39523
RAB5B	Rab5b	14.97	16.08	11.83	11.14	2.57E-05	-1.39746
SLAIN2	Slain2	20.5	20.5	15.66	14.9	3.48E-05	-1.39812
SGK1	Sgk1	5.82	5.66	3.27	4.54	0.005372	-1.39817
Ptbp1	Ptbp1	108.41	100.59	83.29	71.9	4.3E-07	-1.39892
YWHAQ	Ywhaq	170.83	179.3	131.09	132.25	2.38E-06	-1.39895

Gene-ID	Gene Symbol	MG33304	MG33303	MG33306	MG33305	q_value	FC
Fermt2	Fermt2	22.47	22.22	17.93	17.24	8.54E-06	-1.40217
ZC4H2	Zc4h2	2.83	3.42	2.3	2.25	0.020316	-1.40265
TACC1	Tacc1	7.42	7.33	5.68	5.2	0.000185	-1.4035
CD2AP	Cd2ap	17.25	19.65	14.61	12.64	1.99E-05	-1.40552
Gosr2	Gosr2	46.23	45.52	31.46	33.27	2.03E-07	-1.40587
MRFAP1	Mrfap1	236.55	255.57	186.9	181.51	1.25E-07	-1.40657
Socs5	Socs5	3.3	3.18	2.32	2.31	0.005291	-1.40658
CLCN3	Clcn3	12.03	13.42	9.73	9.62	6.64E-05	-1.4074
GNAI3	Gnai3	30.14	33.01	23.58	23.92	1.71E-07	-1.40813
NRP1	Nrp1	81.76	82.29	58.07	57.03	2.82E-06	-1.40824
Atp6v0e	Atp6v0e	220.15	215.89	165.05	161.23	5.54E-08	-1.40875
Zfp148	Zfp148	13.04	14.85	9.95	9.59	3.73E-07	-1.40951
C030046E11Rik	C030046E11Rik	21.27	20.37	16.66	15.35	5.06E-06	-1.41189
UBA6	Uba6	9.44	10.19	7.23	7.21	0.000118	-1.41753
FAM168B	Fam168b	44.72	42.46	33.57	30.78	8.54E-07	-1.41879
TMOD3	Tmod3	11.74	11.42	8.66	8.59	4.98E-06	-1.41968
PSD3	Psd3	8.72	9.77	7.73	6.86	1.13E-05	-1.42059
DCAF7	Dcaf7	19.16	18.53	14.35	12.76	6.98E-06	-1.42082
CTDSPL	Ctdspl	3.15	3.08	2.28	2.06	0.008261	-1.42148
Golga7	Golga7	37.61	36.87	28.92	27.89	4.56E-06	-1.42172
Ube2b	Ube2b	35.38	36.11	28.31	26.29	9.12E-05	-1.4236
CCNG1	Ccng1	127.02	136.4	97.58	97.68	6.23E-08	-1.42456
Rab13	Rab13	17.99	20.99	14.94	12.68	0.000225	-1.4264
MMD	Mmd	38.68	36.74	29.85	28.47	0.000141	-1.42734
Dnajb5	Dnajb5	5.05	4.62	3.45	3.57	0.025743	-1.42834

Gene-ID	Gene Symbol	MG33304	MG33303	MG33306	MG33305	q_value	FC
RAP2B	Rap2b	3.13	3.16	2.64	2.13	0.000895	-1.43084
FUS	Fus	20.92	22.41	15.14	15.44	0.000217	-1.43213
PPFIA1	Ppfia1	15.69	15.9	13.21	11.18	2.53E-05	-1.43227
TAF5L	Taf5l	31.58	30.44	23.66	21.25	2.51E-06	-1.43389
Acaca	Acaca	25.14	24.99	20.07	18.6	1.2E-08	-1.43481
Ppm1f	Ppm1f	16.84	15.58	12.43	11.09	6.67E-07	-1.43562
MBNL1	Mbnl1	40.27	40.97	30.47	29.56	7.23E-07	-1.43594
DPYSL2	Dpysl2	45.56	40.57	34.07	28.66	7.66E-09	-1.43686
TOMM70A	Tomm70a	51.62	55.17	40.37	38.12	3.5E-07	-1.43965
DUSP3	Dusp3	14.69	15.49	10.17	8.96	0.002024	-1.43994
Acap2	Acap2	8.91	9.17	6.51	6.62	2.77E-07	-1.44009
Phf6	Phf6	15.69	16.69	11.92	11.34	9.52E-08	-1.4478
SEC31A	Sec31a	61.08	56.13	43.04	39.22	4.85E-08	-1.4486
CAV2	Cav2	9.19	9.19	7.09	6.96	5.25E-05	-1.44974
VTI1A	Vti1a	6.2	6.14	4.82	4.11	0.010138	-1.44991
Mpdz	Mpdz	34.77	34.98	24.1	22.96	6.02E-08	-1.45086
NAV3	Nav3	1.61	1.56	1	0.71	0.029423	-1.45113
DAG1	Dag1	42.57	40.49	31.62	26.76	2.42E-08	-1.45321
BRMS1L	Brms1l	11.54	13.14	9.45	8.47	5.1E-06	-1.45371
Tmem164	Tmem164	11.98	11.31	8.73	8.04	1.26E-05	-1.45698
UBE2Q1	Ube2q1	25.68	25.62	18.26	18.16	2.23E-08	-1.45817
SSFA2	Ssfa2	11.56	10.81	8.17	7.49	1.86E-07	-1.45855
PRDX1	Prdx1	455.19	486.15	341.27	348.52	2.78E-08	-1.46153
DKC1	Dkc1	51.08	48.21	36.62	34.59	9.38E-07	-1.46335
AP1S2	Ap1s2	9.52	9.96	7.55	7.59	0.000292	-1.46561

Gene-ID	Gene Symbol	MG33304	MG33303	MG33306	MG33305	q_value	FC
Ranbp10	Ranbp10	7.77	7.64	5.65	5.32	4.87E-05	-1.46603
ARPC5	Arpc5	50.08	52.04	38.66	38.7	9.64E-09	-1.46775
Scd2	Scd2	264.6	253.65	201.68	169.61	2.05E-06	-1.46781
RAD23B	Rad23b	102.11	96.54	70.84	67.25	8.56E-10	-1.47109
SNX19	Snx19	18.45	17.59	12.93	12.28	7.21E-07	-1.47473
DSP	Dsp	31.76	32.35	23.78	21.39	2.66E-08	-1.47772
DNAJA1	Dnaja1	134.99	155.82	106.81	100.52	9.22E-07	-1.4788
ACO1	Aco1	27.23	25.96	19.66	17.79	1.8E-09	-1.47942
Ank3	Ank3	15.76	13.87	11.29	10.43	4.8E-06	-1.48139
Golim4	Golim4	10.38	10.1	7.5	6.59	5.47E-06	-1.48342
Tmem120b	Tmem120b	4.52	4.55	3.07	3.51	0.011172	-1.48653
HIPK1	Hipk1	12.26	11.86	8.87	8.24	3.55E-09	-1.48802
ZFX	Zfx	18.16	17.74	13.24	15.02	9.64E-07	-1.48833
Zeb2	Zeb2	20.41	21.14	12.98	11.9	4E-07	-1.49063
GOLPH3	Golph3	55.92	52.92	39.26	37.79	2.13E-10	-1.49182
Sfxn1	Sfxn1	26.15	27.38	19.13	18.41	2.76E-09	-1.49586
ELMOD2	Elmod2	13.4	14.48	12.32	11.69	8.09E-07	-1.49678
Igsf3	Igsf3	5.73	5.83	4.19	3.86	7.56E-06	-1.49695
STX2	Stx2	10.35	9.94	7.71	6.47	8.37E-05	-1.49742
Bnc2	Bnc2	4.29	4.4	3.04	2.81	0.006741	-1.49817
PGK1	Pgk1	645.92	626.42	452.03	446.24	9E-08	-1.49838
N4bp2	N4bp2	5.52	6.38	4.03	3.5	6.05E-05	-1.49931
SLC25A23	Slc25a23	12.11	11.38	7.85	7.08	2.51E-06	-1.50316
CBX1	Cbx1	33	35.8	25.33	22.46	1.01E-06	-1.50532
Vps33a	Vps33a	18.8	17.59	12.84	12.11	2.65E-08	-1.50873

Gene-ID	Gene Symbol	MG33304	MG33303	MG33306	MG33305	q_value	FC
Phc3	Phc3	5.05	4.6	3.95	3.09	0.000006	-1.50928
CREBBP	Crebbp	4.74	4.7	3.49	2.88	0.00001	-1.51067
ZMYM1	Zmym1	10.03	9.53	8.41	6.47	0.046068	-1.52363
CLDN12	Cldn12	21.29	23.21	15.71	15.29	3.24E-08	-1.52401
IDS	Ids	3.82	3.5	2.25	2.68	5.63E-05	-1.52622
KDELR2	Kdelr2	64.75	65.4	45.48	43.76	3.48E-11	-1.52668
LZTR1	Lztr1	82.72	77.2	98.76	80.31	7.67E-07	-1.5273
RAB12	Rab12	26.43	28.14	19.69	17.97	4.39E-09	-1.52783
Mxd4	Mxd4	18.1	18.44	11.7	11.23	9.47E-06	-1.52951
EIF4A2	Eif4a2	89.6	104.93	69.19	71.57	2.14E-07	-1.53
RCHY1	Rchy1	18.9	17.89	12.74	11.66	6.2E-07	-1.53033
LHFP	Lhfp	50.46	50.09	34.01	34.63	3.74E-11	-1.53098
Gjc1	Gjc1	18.18	20.33	13.15	12.16	1.99E-05	-1.53132
CPD	Cpd	25.39	25.74	18.23	16.95	5.96E-12	-1.53201
Tmem41b	Tmem41b	66.74	76.59	50.51	51.98	2.24E-10	-1.5325
Gucy1a3	Gucy1a3	102.99	111.5	75.01	70.41	5.29E-11	-1.53287
SSH1	Ssh1	1.68	1.35	1.06	0.8	0.041712	-1.53321
Atrx	Atrx	47.38	56.08	33.91	37.59	3.32E-08	-1.53672
Ednra	Ednra	6.53	6.98	4.56	4.42	1.69E-06	-1.53749
ARNT	Arnt	11.15	11.41	9.24	8.19	0.000274	-1.53763
SHCBP1	Shcbp1	24.7	28.15	19.06	17.7	1.72E-09	-1.53828
Amotl2	Amotl2	18.91	15.15	11.52	10.25	3.03E-09	-1.54011
Rlf	Rlf	11.38	12.75	8.22	7.88	5.89E-09	-1.54488
Plaa	Plaa	52.82	58.28	38.96	37.61	6.54E-11	-1.54619
IRAK1	Irak1	14.14	12.83	10.9	9.36	1.07E-05	-1.54935

Gene-ID	Gene Symbol	MG33304	MG33303	MG33306	MG33305	q_value	FC
GCLM	Gclm	21.05	20.78	13.71	12.16	0.03359	-1.55043
SLC6A9	Slc6a9	6.75	4.78	5.22	3.47	0.000544	-1.55151
Cggbp1	Cggbp1	42.14	40.7	28.87	28.74	1.41E-10	-1.55248
Glis2	Glis2	6.78	6.57	4.73	4.28	0.002359	-1.55286
SLC30A7	Slc30a7	5.75	5.75	4.05	3.65	4.23E-08	-1.5532
Hccs	Hccs	33.45	33.6	24.01	22.21	1.98E-07	-1.5542
Klf9	Klf9	16.28	17.56	11.1	11.4	2.49E-07	-1.55551
Plk2	Plk2	14.37	14.33	9.49	9.54	9.79E-09	-1.55582
EVI5	Evi5	23.71	25.44	16.85	17.34	5E-10	-1.55764
PALLD	Palld	4.47	4.93	3.42	3.1	0.00028	-1.55879
Zfp292	Zfp292	12.02	15.12	9.11	9	7.83E-12	-1.56228
DCTN3	Dctn3	70.1	63.5	47.12	43.32	5.48E-10	-1.56362
PPP6C	Ppp6c	18.47	20.3	12.12	11.1	2.33E-07	-1.56861
KLHL15	Klhl15	5.33	4.48	3.94	3.19	0.004461	-1.57161
Mecp2	Mecp2	11.43	11.05	7.89	7.38	7.47E-10	-1.5729
NFIX	Nfix	8.34	7.36	5.07	4.23	0.000885	-1.57421
CMPK1	Cmpk1	32.77	36.81	24.4	22.88	5.31E-12	-1.57545
PIK3C2A	Pik3c2a	16.42	19.09	11.59	12.01	1.07E-11	-1.57875
4931406P16Rik	4931406P16Rik	33.74	31.06	21.13	19.32	2.74E-10	-1.58103
LPP	Lpp	9.71	10.3	6.88	6.2	1.52E-05	-1.58565
UBE2E1	Ube2e1	7.91	6.98	4.99	5.23	0.016212	-1.58698
Ripk2	Ripk2	12.39	12.95	8.44	7.89	9.73E-07	-1.58755
Crybg3	Crybg3	16.36	17.34	11.21	11.42	1.97E-12	-1.58786
EIF4EBP2	Eif4ebp2	6.47	5.53	4.48	3.61	2.08E-08	-1.58925
2700081O15Rik	2700081O15Rik	8.13	9.19	6.39	5.64	0.000673	-1.58947

Gene-ID	Gene Symbol	MG33304	MG33303	MG33306	MG33305	q_value	FC
TOMM20	Tomm20	110.82	108.75	69.45	67.5	7.59E-11	-1.59041
SRP72	Srp72	33.01	32.66	22.86	21.7	8.33E-13	-1.59054
SLC7A5	Slc7a5	70.04	59.14	45.53	38.34	6.13E-14	-1.59237
UBE2A	Ube2a	9.89	10.89	7.52	6.57	2.44E-06	-1.59456
Zmym4	Zmym4	12.23	13.63	8.4	8.24	3.42E-06	-1.59559
Pi4kb	Pi4kb	18.16	15.59	11.91	10.18	5.5E-08	-1.59945
Slc35a2	Slc35a2	13.62	11.88	8.46	7.82	0.000473	-1.60064
AMMECR1L	Ammecr1l	6.05	5.49	4	3.64	2.59E-05	-1.60141
ATP6V1A	Atp6v1a	47.32	47.26	32.61	29.7	4.14E-13	-1.60218
GALNT2	Galnt2	98.45	99.94	68.39	62.24	2.39E-13	-1.60273
EGLN1	Egln1	42.02	37.03	27.32	24.24	3.07E-06	-1.60433
BACH1	Bach1	6.08	6.4	4.29	3.78	3.91E-07	-1.60483
Trim2	Trim2	20.83	21.83	13.7	13.54	4.85E-09	-1.60619
Arl8b	Arl8b	38.71	41.91	26.35	26.23	8.39E-14	-1.60826
GCA	Gca	7.6	11.1	7.07	5.27	4.79E-06	-1.61074
Irs1	Irs1	3.98	3.52	2.74	2.48	8.44E-08	-1.61435
A830080D01Rik	A830080D01Rik	6.55	6.85	5.25	3.75	0.000132	-1.61547
Mmgt1	Mmgt1	25.62	28.08	17.22	17.92	5.22E-14	-1.6161
TFG	Tfg	40.2	38.78	25.43	25.15	1.56E-11	-1.61817
XIAP	Xiap	7.43	8.47	5.55	5.29	9.73E-09	-1.6186
Twistnb	Twistnb	38.83	43.53	27.58	26.08	4.93E-11	-1.6193
Sh3pxd2a	Sh3pxd2a	7.33	6.89	4.63	4.38	2.51E-09	-1.61991
RAB31	Rab31	35.73	35.24	23.37	22.86	1.96E-14	-1.62097
TMEM9	Tmem9	39.84	37.12	24.88	24.21	2.69E-10	-1.62282
RAP2C	Rap2c	7.64	7.92	5.33	4.98	3.89E-06	-1.6231

Gene-ID	Gene Symbol	MG33304	MG33303	MG33306	MG33305	q_value	FC
GALC	Galc	5.01	4.56	3.41	3.04	5.88E-07	-1.62367
IMPAD1	Impad1	31.65	33.67	22.66	20.94	4.81E-11	-1.62448
SCHIP1	Schip1	26.25	25.75	16.49	16.6	6.22E-10	-1.6253
TOMM5	Tomm5	141.74	151.53	96.69	92.81	1.5E-11	-1.63273
FNIP1	Fnip1	14.33	17.15	10.3	10.16	5.33E-13	-1.63562
Plekhm3	Plekhm3	2.39	2.63	1.49	1.89	0.005431	-1.6357
ERRFI1	Errfi1	3.94	3.49	2.55	2.17	3.41E-05	-1.63597
Mex3a	Mex3a	3.6	3.57	2.34	2.11	7.18E-05	-1.63658
PAICS	Paics	117.49	118.88	76.48	73.25	5.24E-11	-1.64039
SLC19A2	Slc19a2	21.57	21.29	14.93	13.32	2.54E-12	-1.64701
Prkacb	Prkacb	17.79	16.58	11.9	10.44	2.43E-13	-1.64979
Csrp2	Csrp2	167.97	177.96	109.23	110.02	0	-1.65002
SPRED2	Spred2	2.13	1.71	1.34	1.21	0.042252	-1.65251
Nr2f2	Nr2f2	9.74	9.19	5.54	5.08	1.06E-05	-1.65301
TRAPPC2	Trappc2	12.5	12.4	7.82	8.42	1.31E-05	-1.65854
Plxna4	Plxna4	1.68	1.62	1.57	1.46	0.000223	-1.66056
RAB35	Rab35	25.26	22.67	15.88	13.71	1.2E-12	-1.66082
ERI3	Eri3	37.47	35.56	22.12	21.64	4.5E-09	-1.67369
LDLR	Ldlr	40.2	35.42	25.02	22.04	0	-1.67446
JUN	Jun	2.03	1.34	1.01	1.01	0.005807	-1.67589
RUFY2	Rufy2	5.66	6.13	4.08	3.85	0.000618	-1.6764
LAMP2	Lamp2	103.98	108.62	66.13	66.29	1.02E-11	-1.67751
CYTH3	Cyth3	19.8	19.03	12.07	11.61	2.73E-13	-1.67915
Ikzf2	Ikzf2	1.1	1.2	0.69	0.72	0.001823	-1.68979
Cdk16	Cdk16	35.29	33.88	23.01	21.72	3.84E-14	-1.69075

Gene-ID	Gene Symbol	MG33304	MG33303	MG33306	MG33305	q_value	FC
Mkl2	Mkl2	6.06	6.01	3.86	3.62	5.48E-07	-1.69218
Naa50	Naa50	33.33	33.99	21.61	20.84	4.6E-13	-1.69494
Frmd4a	Frmd4a	4.51	4.72	2.62	2.18	2.74E-05	-1.6966
IKBIP	Ikbip	21.45	22.52	12.85	14.54	2.97E-07	-1.69899
CDR2	Cdr2	15.65	15.82	9.77	9.32	5.38E-12	-1.70075
STAG2	Stag2	46.3	54.01	31.89	31.82	5.11E-15	-1.70334
Dmd	Dmd	7.78	9.14	4.81	4.19	3.64E-05	-1.70396
Hspa13	Hspa13	11.5	12.05	7.49	7.69	8.53E-11	-1.70417
GSPT1	Gspt1	41.69	42.6	27.61	25.77	1.61E-13	-1.7067
STAT5B	Stat5b	11.09	10.45	6.5	6.5	5.52E-11	-1.70987
Psip1	Psip1	87.53	96.23	55.42	55.32	7.25E-12	-1.7109
Itpr1	Itpr1	28.67	28.79	18.38	16.61	0	-1.71192
Rab21	Rab21	35.76	34.02	17.19	15.04	0.000493	-1.71228
Acp1	Acp1	95.3	100.97	59.48	59.12	3.84E-14	-1.71339
Apoo	Apoo	76.49	84.97	53.07	53.84	9.77E-14	-1.71469
Ndst1	Ndst1	23.36	21.15	14.5	12.3	5.86E-08	-1.71634
POLA1	Pola1	41.46	45.01	32.43	30.51	0	-1.71639
DLG1	Dlg1	32.41	33.37	23.17	25.73	1.29E-13	-1.71747
ZBTB33	Zbtb33	8.39	9.24	5.42	5.05	7.72E-09	-1.7286
KDM5B	Kdm5b	6.55	6.41	4.21	3.98	2.86E-11	-1.72932
Lats2	Lats2	6.67	5.83	3.78	3.28	1.97E-07	-1.73043
ZMIZ1	Zmiz1	10.76	9.77	6.62	5.83	1.86E-11	-1.73058
Vbp1	Vbp1	67.95	75.95	43.8	45.05	0	-1.73124
Zfp532	Zfp532	7.66	6.29	4.24	3.67	1.12E-05	-1.73171
VCL	Vcl	126.18	123.17	78.02	71.58	1.66E-11	-1.74043

Gene-ID	Gene Symbol	MG33304	MG33303	MG33306	MG33305	q_value	FC
IFIT2	Ifit2	11.51	14.09	7.05	7.64	1.64E-11	-1.75064
Setd7	Setd7	19.95	21.89	12.93	11.91	0	-1.7511
Ckap4	Ckap4	33.09	31.33	19.45	17.94	0	-1.75975
Heatr5a	Heatr5a	10.61	11.03	6.66	6.16	5.22E-14	-1.76253
Trim62	Trim62	0.94	0.69	0.54	0.39	0.03871	-1.76904
AGPAT9	Agpat9	11.77	12.28	7.43	7.09	5.67E-09	-1.77136
WDR1	Wdr1	154.09	147.22	89.39	85.21	0	-1.77585
INPP5B	Inpp5b	12.02	11.85	7.75	7.04	9.61E-10	-1.77643
CAV1	Cav1	60.28	58.51	34.47	33.86	0	-1.79023
BC030336	BC030336	9.09	9.23	6.4	6.46	1.92E-05	-1.79306
INSIG1	Insig1	166.65	179.17	104.57	98.63	0	-1.79329
SPIN4	Spin4	2.32	2.45	1.49	1.36	1.41E-06	-1.79965
SMC1A	Smc1a	63.69	71.73	37.26	39.48	0	-1.8184
SLC36A1	Slc36a1	4.49	4.77	2.78	2.39	3.9E-08	-1.82
SORT1	Sort1	49.88	51.69	29.61	27.84	0	-1.82339
Arcn1	Arcn1	112.53	118.83	70.52	64.3	0	-1.82662
Tln1	Tln1	39.34	35.31	22.84	21.15	0	-1.84005
OAT	Oat	179.93	173.72	101.65	98.84	0	-1.84154
Ppp1r12b	Ppp1r12b	32.21	30.99	18.82	17.08	0	-1.84404
SPG20	Spg20	27.73	29.99	16.59	16.08	0	-1.84598
Tsc22d1	Tsc22d1	118.64	112.45	65.37	63.94	0	-1.85556
MORC4	Morc4	12.05	12.14	7.47	6.92	6.42E-13	-1.85675
HSP90AB1	Hsp90ab1	257.88	246.79	145.76	131.05	0	-1.87936
COL4A5	Col4a5	61.15	60.3	33.78	32.46	0	-1.88061
DENND4C	Dennd4c	13.43	14.22	8.75	8.28	0	-1.88198

Gene-ID	Gene Symbol	MG33304	MG33303	MG33306	MG33305	q_value	FC
PITPNA	Pitpna	55.09	57.68	32.67	30.91	0	-1.90704
Snx30	Snx30	9.07	9.01	5	4.9	0	-1.90797
CCDC112	Ccdc112	2.01	2.36	1.26	1.12	2.48E-05	-1.9158
ZYG11B	Zyg11b	14.42	15.63	8.24	8.24	0	-1.91817
ANKRD52	Ankrd52	5.94	5.36	3.33	3.18	3.41E-13	-1.9197
CCDC50	Ccdc50	24.36	27.48	16.06	16.46	0	-1.92339
SLC44A1	Slc44a1	12.53	13.64	7.36	6.85	1.4E-12	-1.95433
Mospd2	Mospd2	16.18	17.92	9.53	9.4	6.83E-13	-1.95638
Tmeff2	Tmeff2	3.16	2.93	1.29	1.68	6.66E-07	-1.95828
NDRG1	Ndrg1	8.32	7.23	4.03	3.96	1.04E-12	-1.9659
KLHL29	Klhl29	4.25	3.83	2.31	1.92	4.72E-13	-1.96929
MGST3	Mgst3	35	32.01	18.06	16.83	0	-1.9713
Snx13	Snx13	38.87	44.87	22.39	21.49	0	-2.00464
TMEM64	Tmem64	15.34	14.72	8.23	7.86	0	-2.00562
ANKIB1	Ankib1	27.42	27.76	14.73	14.08	0	-2.01475
Pard3b	Pard3b	10.97	12.01	6.08	6.25	0	-2.02054
TRAM2	Tram2	2.99	2.79	1.16	0.99	0.00041	-2.02362
RUSC2	Rusc2	7.51	6.68	3.55	3.59	1.86E-11	-2.03535
Dcun1d4	Dcun1d4	6.95	6.58	3.55	3.75	4.09E-12	-2.04128
SCARB2	Scarb2	207.52	220.65	109.97	103.41	0	-2.07525
RHOB	Rhob	75.82	64.36	35.96	33.73	0	-2.08137
TUFT1	Tuft1	5.19	5.25	2.62	2.93	1.95E-08	-2.08159
PRELID2	Prelid2	6.29	6.75	3.39	3.06	0.003366	-2.08293
NCEH1	Nceh1	68.8	77.29	37.15	35.86	0	-2.08947
Gpr173	Gpr173	3.38	3.05	2.15	1.89	0.000193	-2.09985

Gene-ID	Gene Symbol	MG33304	MG33303	MG33306	MG33305	q_value	FC
Pik3cb	Pik3cb	6.91	7.05	3.64	3.36	5.11E-15	-2.11808
MARCKSL1	Marcksl1	73.68	67.11	34.39	33.05	0	-2.11902
GNPNAT1	Gnpnat1	5.82	6.94	3.52	3.1	0.000205	-2.15141
Atp11c	Atp11c	10.91	13.17	6.13	5.91	3.83E-10	-2.15405
HYOU1	Hyou1	240.27	217.74	114.26	100.75	0	-2.15651
ARHGEF3	Arhgef3	2.59	2.78	1.32	1.15	8.23E-07	-2.16115
CDC16	Cdc16	38.45	38.82	18.98	18.24	0	-2.18035
PLOD2	Plod2	62.71	64.59	30.71	32.23	0	-2.18216
Kctd10	Kctd10	67.33	62.02	31.36	30.15	0	-2.19595
Arhgap6	Arhgap6	4.59	4.6	2.19	2.2	2.19E-10	-2.21123
CREB3L2	Creb3l2	11.32	9.88	5.04	4.34	0	-2.21712
Prkca	Prkca	14.53	13.29	6.74	6.24	0	-2.22281
Brwd3	Brwd3	2.72	3.17	1.51	1.31	2.51E-11	-2.23024
NFIB	Nfib	42.19	44.53	22.02	19.28	0	-2.25203
CUL4B	Cul4b	39.96	43.25	20.29	19.48	0	-2.26224
Rab21	Rab21	35.76	34.02	17.19	15.04	0	-2.27635
PRMT6	Prmt6	7.85	7.87	3.53	3.6	4.47E-13	-2.29214
SWAP70	Swap70	23.33	21.95	10.71	9.86	0	-2.31066
Prkg1	Prkg1	14.86	14.53	11.86	10.12	1.74E-11	-2.31209
SPATS2L	Spats2l	9.5	9.92	3.59	4.36	3.47E-11	-2.34991
FOXC1	Foxc1	1.3	1.07	0.62	0.47	0.00021	-2.35297
DCBLD2	Dcbld2	47.45	48.7	23.79	22.85	0	-2.3683
Pak3	Pak3	6.16	6.13	2.48	2.11	2.05E-07	-2.36897
ZFHX3	Zfhx3	4.08	3.99	1.82	1.65	0	-2.37265
ADCY9	Adcy9	2.01	1.84	0.96	0.95	4.01E-06	-2.38993

Gene-ID	Gene Symbol	MG33304	MG33303	MG33306	MG33305	q_value	FC
POLE3	Pole3	27.77	28.99	10.65	10.04	1.78E-12	-2.41257
STX17	Stx17	8.94	9.91	4.31	3.99	5.69E-14	-2.41619
Slc30a4	Slc30a4	17.34	18.84	8.25	7.59	0	-2.41631
Fhod1	Fhod1	6.27	5.87	3.58	2.59	0	-2.46414
Nuak1	Nuak1	5.11	4.55	1.98	1.79	3.84E-14	-2.49591
Rgl1	Rgl1	14.4	13.83	6.23	5.58	0	-2.49685
UBASH3B	Ubash3b	3.21	2.49	1.44	0.99	1.73E-08	-2.52443
Akt3	Akt3	8.69	10.55	3.55	3.53	0	-2.53254
Fam120c	Fam120c	3.42	3.41	1.58	1.44	0	-2.53544
Cadm1	Cadm1	11.88	10.99	5.75	4.4	0	-2.55293
Fgd1	Fgd1	10.5	8.82	4.32	3.79	0	-2.55359
Nrip1	Nrip1	3.14	3.62	1.52	1.3	7.85E-12	-2.5689
RAB11FIP1	Rab11fip1	11.43	10.32	4.43	4.1	0	-2.64192
CNN3	Cnn3	124.81	118	48.86	45.21	0	-2.66303
Pfn2	Pfn2	37.29	36.47	14.83	14.85	0	-2.66453
EIF5A2	Eif5a2	18.22	20.32	8.09	7.44	0	-2.67225
Klf3	Klf3	3.95	3.37	1.51	1.4	8.26E-13	-2.68352
Rps6ka3	Rps6ka3	11.29	11.46	4.91	4.5	0	-2.71827
LRRC58	Lrrc58	14.7	14.27	6.33	5.42	0	-2.72137
Zfp704	Zfp704	2.76	2.88	1.16	0.95	0	-2.73114
Mtss1l	Mtss1l	4.01	3.7	1.69	1.25	1.16E-10	-2.75063
LIMA1	Lima1	11.24	11.2	4.38	3.94	0	-2.75722
Gem	Gem	8.82	9.48	2.92	3.69	0	-2.84983
DUSP6	Dusp6	7.74	6.84	3.06	2.4	0	-2.877
Efna1	Efna1	14.96	15.39	5.59	5.07	0	-2.88808

Gene-ID	Gene Symbol	MG33304	MG33303	MG33306	MG33305	q_value	FC
TM4SF1	Tm4sf1	13.66	12.6	4.53	5.05	0	-2.94195
Nrp2	Nrp2	49.35	49.03	17.63	16.25	0	-3.03079
Xkr4	Xkr4	0.48	0.61	0.24	0.19	0.002415	-3.09353
SRPX	Srpx	11.51	10.13	3.33	3.76	0	-3.18586
Frmd4b	Frmd4b	2.4	2.1	1.01	1.75	1.05E-06	-3.19252
Slc4a4	Slc4a4	16.48	15.76	5.54	4.65	0	-3.27047
PLS3	Pls3	26.38	27.43	8.17	8.01	0	-3.44485
PSD3	Psd3	8.72	9.77	7.73	6.86	0.023699	-3.45772
ABCA1	Abca1	36.71	35.49	11.15	10.21	0	-3.63669
Plcl1	Plcl1	1.46	1.4	0.42	0.43	2.27E-11	-3.64586
MAMLD1	Mamld1	2.48	2.23	0.78	0.53	1.91E-09	-3.7578
FSTL1	Fstl1	257.3	257.88	72.93	72.18	0	-3.79234
SERPINE1	Serpine1	7.24	6.13	2	1.59	0	-3.92088
DLG5	Dlg5	6.77	6.38	1.85	1.02	0	-4.0172
Prkar2b	Prkar2b	5.41	6.05	1.44	1.45	0	-4.05181
LPAR1	Lpar1	4.77	4.45	1.25	0.98	0	-4.12137
ETV5	Etv5	8.18	7.12	1.99	1.81	0	-4.1448
Col4a3	Col4a3	22.91	23.71	6	5.02	0	-4.33234
Fam107b	Fam107b	12.99	14.14	3.13	3.35	0	-4.37158
CLTA	Clta	221.41	208.5	52.57	48.56	0	-4.40659
HS3ST3B1	Hs3st3b1	3.84	3.4	0.94	0.81	0	-4.44233
Nxph1	Nxph1	2.25	2.5	0.64	0.51	4.04E-07	-4.60035
RUNX1	Runx1	5.33	5.11	1.24	1.01	0	-4.89122
Mras	Mras	7.55	8.11	1.61	1.87	0	-4.98725
Cyp1b1	Cyp1b1	1.03	1.2	0.21	0.18	2.38E-10	-5.18351

Gene-ID	Gene Symbol	MG33304	MG33303	MG33306	MG33305	q_value	FC
KDR	Kdr	2.67	2.57	0.53	0.53	0	-5.20678
Hs3st1	Hs3st1	3.23	2.87	0.29	0.37	0	-5.50743
SMARCA1	Smarca1	7.53	8.33	1.14	1.6	0	-5.68076
Ppap2b	Ppap2b	22.11	20.83	4.17	3.57	0	-5.79803
CDK5R1	Cdk5r1	10.35	8.94	1.75	1.74	0	-5.80345
Rasgef1b	Rasgef1b	11.89	12.37	2.42	2	0	-5.99168
ADM	Adm	142.69	133.88	22.8	21.43	0	-6.421
IGFBP4	Igfbp4	53.38	48.95	10.1	9.4	0	-7.78608
Reck	Reck	6.19	5.09	0.78	0.71	0	-8.12236
Inhba	Inhba	8.38	7.82	0.9	1.08	0	-8.91328
ANXA1	Anxa1	0.67	0.73	0.3	0.11	0.000566	-9.22248
Kank1	Kank1	2.87	2.73	0.32	0.37	0	-10.5403
MYOF	Myof	5.7	5.39	0.58	0.5	0	-10.6167
Rims3	Rims3	1.01	0.83	0.09	0.08	1.02E-13	-10.6999
PEG10	Peg10	11.18	9.84	1.15	1.08	0	-10.9477
THBS1	Thbs1	0.64	0.56	0.06	0.05	2.99E-10	-17.8662
Adamts8	Adamts8	13.8	11.29	0.61	0.33	0	-28.3337
Fat3	Fat3	3.46	3.48	0.11	0.13	0	-29.2762
PMEPA1	Pmepa1	4.79	4.95	0.13	0.06	3.03E-08	-388.792

Supplementary Table 2. miR-200 family highly influence focal adhesion pathway. Pathways analysis of miR-200 family targets genes showed a remarkable influence of miR-200 family to the focal adhesion pathway.

p-value	q-value	pathway	source	external_id	members_input_overlap	members_input_overlap_geneids	size	effective_size
2,51E-10	8,42E-09	Membrane Trafficking	Reactome	R-HSA-199991	CTTN; PUM1; DENND5A; PRKAG2; CPD; KIF26B; AKT3; CLTA; AP1S2; AP1S3; DCTN3; CHMP5; RAB8B; RAB35; SEC23A; RAB31; STAM2; YWHAQ; PPP6C; SEC31A; RABEP1; RAB13; RAB12; DENND4C; RAB5B; ARPC5; KDELR2; PIK3C2A; SEC24A; DYNC1LI2; GDI2;	23011; 130340; 5286; 5869; 10092; 9698; 90411; 288; 51762; 950; 1362; 10000; 9265; 6399; 9135; 6272; 5537; 10254; 2590; 5872; 10971; 51422; 55014; 8905; 3267; 11021; 10342; 201475; 51272; 1783; 55667; 10484; 2017; 2665; 10802; 372; 27333; 11014; 51510; 64746; 55083; 143187; 9570; 23258; 3949; 1211; 22872; 11031; 11258	580	579

					MCFD2; CYTH3; GOSR2; GALNT2; RAB21; BET1L; SCARB2; TFG; TRAPPC2; LDLR; GOLIM4; ARCN1; ACBD3; ANK3; VTI1A; STX17; SORT1; AGFG1			
2,55E-10	8,42E-09	Vesicle-mediated transport	Reactome	R-HSA-5653656	CTTN; PUM1; DENND5A; PRKAG2; CPD; KIF26B; AKT3; CLTA; AP1S2; AP1S3; DCTN3; CHMP5; RAB8B; RAB35; GOLIM4; HSPH1; RAB31; HYOU1; STAM2; YWHAQ; PPP6C; SEC31A; RABEP1; RAB13; RAB12; DENND4C; RAB5B; ARPC5; KDELR2;	23011; 5286; 5869; 10092; 9698; 201475; 90411; 288; 10802; 950; 10000; 9265; 6399; 9135; 6272; 5537; 10254; 1362; 2590; 10971; 130340; 51422; 55014; 8905; 3267; 11021; 10342; 3949; 10808; 51272; 55667; 1783; 10484; 2017; 2665; 51762; 372; 27333; 11014; 51510; 64746; 55083; 143187; 9570; 23258;	619	618

					PIK3C2A; SEC24A; DYNC1LI2; GDI2; MCFD2; CYTH3; GOSR2; GALNT2; SEC23A; RAB21; BET1L; SCARB2; TFG; TRAPPC2; LDLR; ARCN1; ACBD3; ANK3; VTI1A; STX17; SORT1; AGFG1	5872; 1211; 22872; 10525; 11031; 11258		
4,18E-09	9,20E-08	Focal adhesion - Homo sapiens (human)	KEGG	path:hsa04510	CRKL; RAP1B; XIAP; AKT3; CRK; ARHGAP5; THBS1; CAV2; CAV1; PIK3CB; JUN; TLN1; CAPN2; PIK3R1; KDR; IGF1R; PPP1R12B; PRKCA; VCL; COL4A5; COL4A3; PPP1CC;	1398; 4660; 5728; 7094; 5908; 3725; 5291; 5295; 3791; 7414; 1285; 1287; 10000; 824; 857; 858; 2932; 1399; 5501; 394; 7057; 3480; 331; 5063; 5578	201	200

					GSK3B; PTEN; PAK3,ITGA1			
1,27E-06	2,09E-05	Neurotrophin signaling pathway - Homo sapiens (human)	KEGG	path:hsa04722	RPS6KA3; CRKL; PTPN11; JUN; IRS1; FRS2; AKT3; SORT1; RIPK2; RAP1B; GSK3B; PIK3R1; IRAK2; CRK; IRAK1; PIK3CB	6197; 8767; 10818; 3654; 3656; 3667; 6272; 3725; 5781; 5291; 5295; 10000; 5908; 2932; 1398; 1399	121	121
2,02E-06	2,33E-05	Signaling by PDGF	Reactome	R-HSA-186797	CRKL; RAP1B; SPRED2; FRS2; PRKAR2B; CRK; THBS1; IQGAP1; IRS1; PIK3CB; TLN1; PIK3R1; DUSP6; PIP4K2B; PTEN; PRKCA; VCL; SPRED1; COL4A5; COL4A3; ITPR1; PTPN12; PTPN11;	3667; 5567; 5578; 5577; 115; 1848; 10818; 200734; 7057; 1398; 5295; 5782; 5781; 8396; 5908; 5728; 5291; 7414; 1399; 8826; 6777; 7094; 2932; 161742; 1287; 10000; 3708; 1285	331	330

					ADCY9; GSK3B; STAT5B; AKT3; PRKACB			

p-value	q-value	pathway	source	external_id	members_input_overlap	members_input_overlap_geneids	size	effective_size
2,12E-06	2,33E-05	Proteoglycans in cancer - Homo sapiens (human)	KEGG	path:hsa05205	IGF1R; CTTN; PIK3R1; ANK3; PIK3CB; THBS1; PTPN11; PPP1CC; RPS6KB1; MRAS; EZR; FRS2; PRKCA; AKT3; PPP1R12B; PRKACB; ITPR1; CAV2; IQGAP1; KDR; CAV1	7430; 4660; 6198; 10818; 10000; 8826; 3708; 5781; 5291; 5295; 3791; 5567; 22808; 288; 857; 858; 5501; 7057; 3480; 5578; 2017	205	205
1,57E-05	0,000148	Signalling by NGF	Reactome	R-HSA-166520	CRKL; SPRED1; SPRED2; FRS2; AKT3; CLTA; CRK; IQGAP1; PIK3CB; ARHGEF3; TLN1; ADCY9; PIK3R1; IRAK1; DUSP3;	5578; 1848; 9828; 200734; 5291; 8826; 8767; 7094; 10000; 3708; 3667; 5567; 5577; 1398; 5728; 6197; 2245; 7414; 8396; 10818; 161742; 3654; 1399; 1845;	433	429

					PIP4K2B; FGD1; ARHGEF17; PRKCA; VCL; RAP1B; PRKAR2B; ITPR1; RPS6KA3; DUSP6; PTPN11; IRS1; GSK3B; PTEN; RIPK2; PRKACB	50650; 5295; 5781; 5908; 1211; 2932; 115		
2,14E-05	0,000169	ER to Golgi Anterograde Transport	Reactome	R-HSA-199977	SEC23A; TFG; TRAPPC2; ARCN1; SEC24A; GOSR2; ANK3; PPP6C; STX17; DYNC1LI2; DCTN3; KDELR2; MCFD2; BET1L; SEC31A	5537; 11014; 10484; 51272; 1783; 90411; 9570; 288; 55014; 22872; 10802; 372; 10342; 6399; 11258	134	134
2,69E-05	0,000169	mTOR signaling pathway - Homo sapiens (human)	KEGG	path:hsa04150	ULK2; FNIP1; IGF1R; RPS6KA3; ATP6V1A; SGK1; PRKCA; PIK3CB; SLC7A5; RPS6KB1; IRS1;	523; 6197; 6198; 3667; 5728; 5291; 96459; 10000; 6446; 5295; 2932; 6009; 3480; 5578; 8140; 9706	154	153

					GSK3B; PTEN; AKT3; PIK3R1; RHEB			

p-value	q-value	pathway	source	external_id	members_input_overlap	members_input_overlap_geneids	size	effective_size
2,73E-05	0,000169	Axon guidance	Reactome	R-HSA-422475	RAP1B; SPRED2; FRS2; CLTA; GSK3B; PITPNA; IQGAP1; DLG1; TLN1; HSP90AB1; CDK5R1; DUSP6; KDR; DPYSL2; PRKCA; VCL; EFNA1; SPRED1; COL4A5; COL4A3; ARPC5; RHOB; NRP1; NRP2; RPS6KA3; PTPN11; IRS1; EZR; PLXNA4; PFN2; ANK3; PRKACB; PAK3	5578; 7430; 1848; 1739; 10092; 200734; 1287; 8829; 288; 8826; 7094; 1808; 1942; 3667; 5567; 3326; 1285; 6197; 5063; 5306; 5217; 10818; 388; 8828; 3791; 91584; 8851; 5781; 5908; 7414; 1211; 2932; 161742	487	484

2,82E-05	0,000169	NGF signalling via TRKA from the plasma membrane	Reactome	R-HSA-187037	CRKL; RAP1B; SPRED2; FRS2; PRKAR2B; CLTA; CRK; GSK3B; IQGAP1; PIK3CB; TLN1; PIK3R1; DUSP6; DUSP3; PIP4K2B; PRKCA; VCL; SPRED1; ITPR1; RPS6KA3; PTPN11; IRS1; ADCY9; PTEN; AKT3; PRKACB	3667; 5567; 5578; 8396; 1399; 5577; 1845; 5728; 1848; 10818; 200734; 115; 5781; 1398; 5295; 1211; 5908; 6197; 5291; 7414; 8826; 7094; 2932; 161742; 10000; 3708	340	339
3,60E-05	0,000198	Insulin signaling pathway - Homo sapiens (human)	KEGG	path:hsa04910	PRKAG2; ACACA; PPP1CC; CRKL; PIK3CB; MKNK1; RPS6KB1; IRS1; GSK3B; AKT3; PIK3R1; PRKACB; PRKAR2B; CRK; RHEB	31; 6198; 3667; 5291; 5295; 8569; 51422; 10000; 2932; 1398; 1399; 6009; 5501; 5567; 5577	140	140

4,00E-05	0,000203	Downstream signal transduction	Reactome	R-HSA-186763	CRKL; RAP1B; SPRED2; FRS2; PRKAR2B; CRK; IQGAP1; IRS1; PIK3CB; TLN1; PIK3R1; DUSP6; PIP4K2B; PTEN; PRKCA; VCL; SPRED1; ITPR1; PTPN11; ADCY9; GSK3B; STAT5B; AKT3; PRKACB	3667; 5567; 5578; 5577; 115; 1848; 10818; 200734; 1398; 5295; 5781; 8396; 5908; 5728; 5291; 7414; 1399; 8826; 6777; 7094; 2932; 161742; 10000; 3708	307	306
7,37E-05	0,000348	Signaling by EGFR	Reactome	R-HSA-177929	RAP1B; SPRED2; FRS2; AKT3; IQGAP1; IRS1; STAM2; TLN1; PIK3R1; DUSP6; PIP4K2B; PRKCA; VCL; SPRED1; ITPR1; LRIG1; PTPN12; PTPN11; ADCY9; GSK3B; PTEN; PRKAR2B; PRKACB; PIK3CB	3667; 5567; 5578; 5577; 115; 5728; 10254; 1848; 10818; 200734; 5295; 5782; 5781; 8396; 5908; 5291; 7414; 8826; 7094; 2932; 161742; 10000; 3708; 26018	319	318

0,000137	0,000599	Rap1 signaling pathway - Homo sapiens (human)	KEGG	path:hsa04015	MRAS; LPAR1; IGF1R; CRKL; PRKCA; PIK3CB; ADCY9; EFNA1; TLN1; PFN2; AKT3; RAP1B; PIK3R1; CRK; THBS1; GNAI3; KDR; PRKD3	5291; 7094; 5217; 115; 23683; 5295; 3791; 10000; 5908; 22808; 2773; 7057; 1902; 1398; 1399; 3480; 1942; 5578	212	212
0,000145	0,000599	Regulation of actin cytoskeleton - Homo sapiens (human)	KEGG	path:hsa04810	ARPC5; FGD1; PIK3R1; CRKL; EZR; PIK3CB; PPP1CC; VCL; MRAS; GNG12; PFN2; CFL2; PPP1R12B; SSH1; CRK; PAK3; IQGAP1; PIP4K2B	5217; 1073; 7430; 4660; 8826; 55970; 5291; 5295; 2245; 8396; 22808; 10092; 1398; 1399; 5501; 54434; 7414; 5063	214	213
0,000154	0,000599	Pathways in cancer - Homo sapiens (human)	KEGG	path:hsa05200	CRKL; XIAP; AKT3; EDNRA; CRK; GNAI3; PTEN; PIK3CB; JUN; GNG12; HSP90AB1; PIK3R1; IGF1R; PRKCA;	10342; 115; 331; 405; 6777; 55970; 2773; 54583; 861; 2932; 5291; 5295; 1285; 1287; 1387; 1398; 1399; 3480; 5567; 5578;	397	397

					COL4A5; COL4A3; EGLN1; ARNT; TFG; ADCY9; GSK3B; STAT5B; RUNX1; PRKACB; CCDC6; LPAR1; CREBBP	3326; 5728; 3725; 10000; 8030; 1902; 1909		

p-value	q-value	pathway	source	external_id	members_input_overlap	members_input_overlap_geneids	size	effective_size
0,000226	0,000752	Endocytosis - Homo sapiens (human)	KEGG	path:hsa04144	RAB35; IGF1R; RAB31; SPG20; ACAP2; STAM2; RAB5B; RAB11FIP1; ARPC5; PSD3; AGAP1; LDLR; CLTA; CHMP5; RABEP1; CYTH3; CAV2; ASAP1; KDR; CAV1	10254; 857; 9265; 23111; 50807; 1211; 3791; 5869; 11021; 11031; 23362; 51510; 858; 80223; 10092; 3949; 3480; 9135; 116987; 23527	260	260
0,000233	0,000752	Transport to the Golgi and	Reactome	R-HSA-948021	SEC23A; TFG; TRAPPC2; ARCN1; SEC24A; GOSR2; ANK3; PPP6C; STX17;	5537; 11014; 10484; 51272; 1783; 90411; 9570; 288; 55014;	165	165

		subsequent modification			DYNC1LI2; DCTN3; KDELR2; MCFD2; BET1L; SEC31A	22872; 10802; 372; 10342; 6399; 11258		
0,000233	0,000752	Protein processing in endoplasmic reticulum - Homo sapiens (human)	KEGG	path:hsa04141	BAG2; SEL1L; DNAJA1; RAD23B; HSPH1; HSPA5; HYOU1; SEC31A; HSP90AB1; SEC24A; CAPN2; EDEM1; PLAA; CKAP4; SEC23A	10802; 9532; 10808; 9373; 10970; 3301; 3309; 10484; 3326; 5887; 6400; 10525; 824; 22872; 9695	166	165
0,000239	0,000752	Post-translational protein modification	Reactome	R-HSA-597592	RECK; UBE2B; RAD23B; CLSPN; SMAD7; MAP3K7; RIPK2; RHOT1; DCTN3; USP25; SEC31A; RAB8B; RAB35; SEC23A; RAB31; STAM2; ADAMTS8; THBS1; PPP6C; PHC3; PTP4A2;	23011; 1605; 4092; 5277; 5869; 11095; 10735; 29761; 90411; 288; 5872; 6885; 51762; 8767; 5537; 10484; 10254; 2590; 7057; 80012; 55288; 5728; 55014; 6399; 63967; 55236; 8073; 11021; 10342; 142; 201475; 51272; 1783; 7324; 56648; 6400;	1026	1025

					RAB13; RAB12; SMC1A; PIGA; SEL1L; GNPNAT1; RAB5B; STAG2; SENP5; UBE2E1; PARP1; UBA6; GOSR2; SEC24A; DAG1; DYNC1LI2; MCFD2; GALNT2; UBE2I; RAB21; UBE2K; BET1L; UBE2A; TFG; TRAPPC2; ARCN1; PTEN; ANK3; STX17; KDELR2; EIF5A2; TOMM20	10802; 372; 8434; 11014; 7319; 64841; 8243; 9570; 205564; 3093; 7320; 9804; 7329; 22872; 5887; 11031; 11258		
0,000345	0,001036	Signaling by VEGF	Reactome	R-HSA-194138	IQGAP1; ITPR1; PRKCA; PIK3CB; VCL; IRS1; SPRED2; FRS2; PAK3; AKT3; RAP1B; TLN1; PIK3R1; PRKACB; NRP2;	3667; 5567; 5578; 3791; 3708; 1848; 10818; 200734; 1398; 5295; 857; 8829; 5908; 5291; 7414; 8826; 7094; 161742; 10000; 8828; 5063	290	289

					CRK; DUSP6; SPRED1; NRP1; KDR; CAV1			
0,000865	0,002482	DAP12 signaling	Reactome	R-HSA-2424491	IQGAP1; ITPR1; PRKCA; VCL; PTPN11; GSK3B; FRS2; ADCY9; SPRED2; IRS1; AKT3; PTEN; PRKAR2B; RAP1B; TLN1; PIK3R1; PRKACB; DUSP6; SPRED1; PIK3CB; PIP4K2B	3667; 5567; 5578; 5577; 115; 1848; 10818; 200734; 5295; 5781; 8396; 5908; 5728; 5291; 7414; 8826; 7094; 2932; 161742; 10000; 3708	311	310
0,001197	0,003292	PI3K-Akt signaling pathway - Homo sapiens (human)	KEGG	path:hsa04151	AKT3; MCL1; PIK3CB; YWHAQ; RPS6KB1; GNG12; THBS1; HSP90AB1; PIK3R1; KDR; IGF1R; PRKCA; EFNA1; COL4A5; COL4A3; RHEB;	1285; 3326; 6198; 5728; 4170; 3667; 55970; 2932; 3791; 6009; 1287; 10000; 6446; 10971; 7057; 1902; 64764; 1942; 3480; 5578; 5291; 5295	341	340

					SGK1; IRS1; GSK3B; CREB3L2; PTEN; LPAR1			
0,001318	0,00336	Developmental Biology	Reactome	R-HSA-1266738	RAP1B; SPRED2; FRS2; AKT3; CLTA; GSK3B; PITPNA; IQGAP1; DLG1; TLN1; HSP90AB1; TSC22D1; MED13; CDK5R1; DUSP6; KDR; DPYSL2; PRKCA; TCF12; VCL; EFNA1; SPRED1; COL4A5; COL4A3; ARPC5; RHOB; NRP1; NRP2; RPS6KA3; PTPN11; IRS1; EZR; PLXNA4; DSP; PFN2; ANK3; PRKACB; CREBBP; PAK3	5578; 1848; 1739; 10092; 200734; 8829; 288; 8826; 1808; 7094; 7430; 1942; 6938; 3667; 5567; 8848; 10000; 3326; 1285; 6197; 5908; 1287; 5063; 5306; 5217; 10818; 388; 1387; 8828; 9969; 3791; 91584; 8851; 1832; 5781; 7414; 1211; 2932; 161742	748	745

0,001323	0,00336	Metabolism of proteins	Reactome	R-HSA-392499	RECK; UBE2B; RAD23B; VBP1; HSPA5; CLSPN; SMAD7; SEL1L; ADAMTS8; RIPK2; RHOT1; DCTN3; USP25; THBS1; SLC30A7; SEC31A; RAB8B; RAB35; TOMM5; PMPCB; RAB31; STAM2; FKBP9; MAP3K7; GNG12; GNAI3; INHBA; DYNC1LI2; PPP6C; PHC3; PTP4A2; RAB13; BET1L; SMC1A; PIGA; GSPT1; GNPNAT1; RAB5B; STAG2; RAB12; SENP5; UBE2E1; PARP1; UBA6;	23011; 29761; 64841; 6885; 401505; 6399; 10254; 3093; 51272; 56648; 1974; 8434; 3309; 142; 22872; 5887; 9512; 5869; 11095; 55970; 5537; 63967; 11021; 7411; 3624; 7319; 9570; 10970; 148867; 11031; 10802; 10484; 2590; 5728; 6731; 8073; 10342; 2773; 201475; 372; 55288; 3487; 11014; 288; 8243; 7320; 9804; 1605; 4092; 5277; 10735; 90411; 10238; 8767; 5872; 7057; 80012; 55014; 55236; 2935; 1783; 7324; 11328; 6400; 51762; 7329; 205564; 11258	1506	1502

					KDELR2; SEC24A; DAG1; IGFBP4; MCFD2; GALNT2; SEC23A; UBE2I; RAB21; UBE2K; SRP72; UBE2A; TFG; TRAPPC2; ARCN1; TOMM20; PTEN; ANK3; DCAF7; STX17; GOSR2; EIF5A2; CKAP4; EIF4A2			

p-value	q-value	pathway	source	external_id	members_input_overlap	members_input_overlap_geneids	size	effective_size
0,00143	0,003496	HTLV-I infection - Homo sapiens (human)	KEGG	path:hsa05166	DLG1; CDC16; BUB3; PIK3CB; JUN; ADCY9; MRAS; STAT5B; XIAP; AKT3; POLE3; TLN1; PIK3R1; PRKACB;	5295; 1739; 10000; 115; 6777; 8829; 3725; 5291; 8881; 1387; 22808; 54107; 2932; 7094; 5567; 331; 9184; 4603	258	258

					MYBL1; GSK3B; NRP1; CREBBP			
0,001536	0,003557	VEGFA-VEGFR2 Pathway	Reactome	R-HSA-4420097	ITPR1; PRKCA; PIK3CB; VCL; IRS1; SPRED2; FRS2; PAK3; AKT3; RAP1B; TLN1; PIK3R1; PRKACB; CRK; DUSP6; SPRED1; IQGAP1; KDR; CAV1	3667; 5567; 5578; 3791; 1848; 10818; 200734; 1398; 5295; 857; 5908; 5291; 7414; 8826; 7094; 161742; 10000; 3708; 5063	282	281
0,001563	0,003557	DAP12 interactions	Reactome	R-HSA-2172127	IQGAP1; ITPR1; PRKCA; VCL; PTPN11; GSK3B; FRS2; ADCY9; SPRED2; IRS1; AKT3; PTEN; PRKAR2B; RAP1B; TLN1; PIK3R1; PRKACB; DUSP6; SPRED1; PIK3CB; PIP4K2B	3667; 5567; 5578; 5577; 115; 1848; 10818; 200734; 5295; 5781; 8396; 5908; 5728; 5291; 7414; 8826; 7094; 2932; 161742; 10000; 3708	326	325

0,003897	0,008298	Asparagine N-linked glycosylation	Reactome	R-HSA-446203	SEL1L; GNPNAT1; SEC23A; TFG; TRAPPC2; RAD23B; ARCN1; SEC24A; KDELR2; ANK3; PPP6C; STX17; DYNC1LI2; DCTN3; GOSR2; MCFD2; BET1L; SEC31A	5537; 51272; 10484; 1783; 64841; 6399; 90411; 9570; 288; 55014; 6400; 22872; 10802; 372; 10342; 11014; 5887; 11258	283	283
0,003897	0,008298	Platelet activation, signaling and aggregation	Reactome	R-HSA-76002	ITPR1; GNAI3; WDR1; HSPA5; PRKCA; PTPN11; VCL; RAP1B; GNG12; THBS1; AKT3; PIK3CB; LAMP2; PIK3R1; CRK; RHOB; TLN1; SERPINE1	7414; 388; 5578; 3309; 3920; 7057; 5054; 1398; 55970; 5295; 2773; 5781; 5908; 5291; 9948; 7094; 10000; 3708	283	283
0,0052	0,010573	Signaling by SCF-KIT	Reactome	R-HSA-1433557	IQGAP1; STAT5B; PRKCA; PIK3CB; GSK3B; VCL; IRS1; SPRED2;	3667; 5578; 1848; 8396; 10818; 200734; 5728; 5295; 5908; 5291;	292	291

					FRS2; PTEN; AKT3; RAP1B; TLN1; PIK3R1; DUSP6; SPRED1; PTPN11; PIP4K2B	7414; 5781; 8826; 6777; 7094; 2932; 161742; 10000		
0,005286	0,010573	Interleukin-3, 5 and GM-CSF signaling	Reactome	R-HSA-512988	CRKL; IQGAP1; PIK3CB; VCL; IRS1; SPRED2; FRS2; STAT5B; RAP1B; TLN1; PIK3R1; CRK; DUSP6; SPRED1; PTPN11	3667; 1399; 1848; 10818; 200734; 1398; 5295; 5908; 5291; 7414; 5781; 8826; 6777; 7094; 161742	226	225
0,00596	0,01157	Ras signaling pathway - Homo sapiens (human)	KEGG	path:hsa04014	IGF1R; RAB5B; RGL1; PIK3CB; RAP1B; GNG12; MRAS; PRKCA; AKT3; EFNA1; PIK3R1; PRKACB; PTPN11; KDR; PAK3	10000; 5908; 23179; 5781; 55970; 5291; 3791; 22808; 5295; 5869; 1942; 3480; 5567; 5063; 5578	229	228
0,006422	0,011774	IRS-related events	Reactome	R-HSA-2428928	IGF1R; RHEB; IQGAP1; VCL; PTPN11; PRKAG2; RPS6KB1; IRS1; SPRED2;	3667; 1848; 10818; 6009; 200734; 51422; 5781; 6198;	254	252

		triggered by IGF1R			FRS2; RAP1B; TLN1; PIK3R1; DUSP6; SPRED1; PIK3CB	5908; 5291; 7414; 5295; 8826; 7094; 161742; 3480		
0,006422	0,011774	IGF1R signaling cascade	Reactome	R-HSA-2428924	IGF1R; RHEB; IQGAP1; VCL; PTPN11; PRKAG2; RPS6KB1; IRS1; SPRED2; FRS2; RAP1B; TLN1; PIK3R1; DUSP6; SPRED1; PIK3CB	3667; 1848; 10818; 6009; 200734; 51422; 5781; 6198; 5908; 5291; 7414; 5295; 8826; 7094; 161742; 3480	254	252

p-value	q-value	pathway	source	external_id	members_input_overlap	members_input_overlap_geneids	size	effective_size
0,006664	0,011886	Signaling by Type 1 Insulin-like Growth Factor 1 Receptor (IGF1R)	Reactome	R-HSA-2404192	IGF1R; RHEB; IQGAP1; VCL; PTPN11; PRKAG2; RPS6KB1; IRS1; SPRED2; FRS2; RAP1B; TLN1; PIK3R1; DUSP6; SPRED1; PIK3CB	3667; 1848; 10818; 6009; 200734; 51422; 5781; 6198; 5908; 5291; 7414; 5295; 8826; 7094; 161742; 3480	255	253

0,007497	0,013021	Signaling by Interleukins	Reactome	R-HSA-449147	SOCS5; IRAK1; FBXW11; CRKL; IRS1; IQGAP1; VCL; PIK3CB; FRS2; MAP3K7; SPRED2; TLN1; STAT5B; RIPK2; RAP1B; PIK3R1; IRAK2; CRK; DUSP6; SPRED1; PTPN11	1848; 200734; 6885; 5291; 8767; 7094; 3667; 3656; 1398; 6777; 10818; 7414; 3654; 1399; 23291; 5295; 9655; 5781; 5908; 8826; 161742	373	372

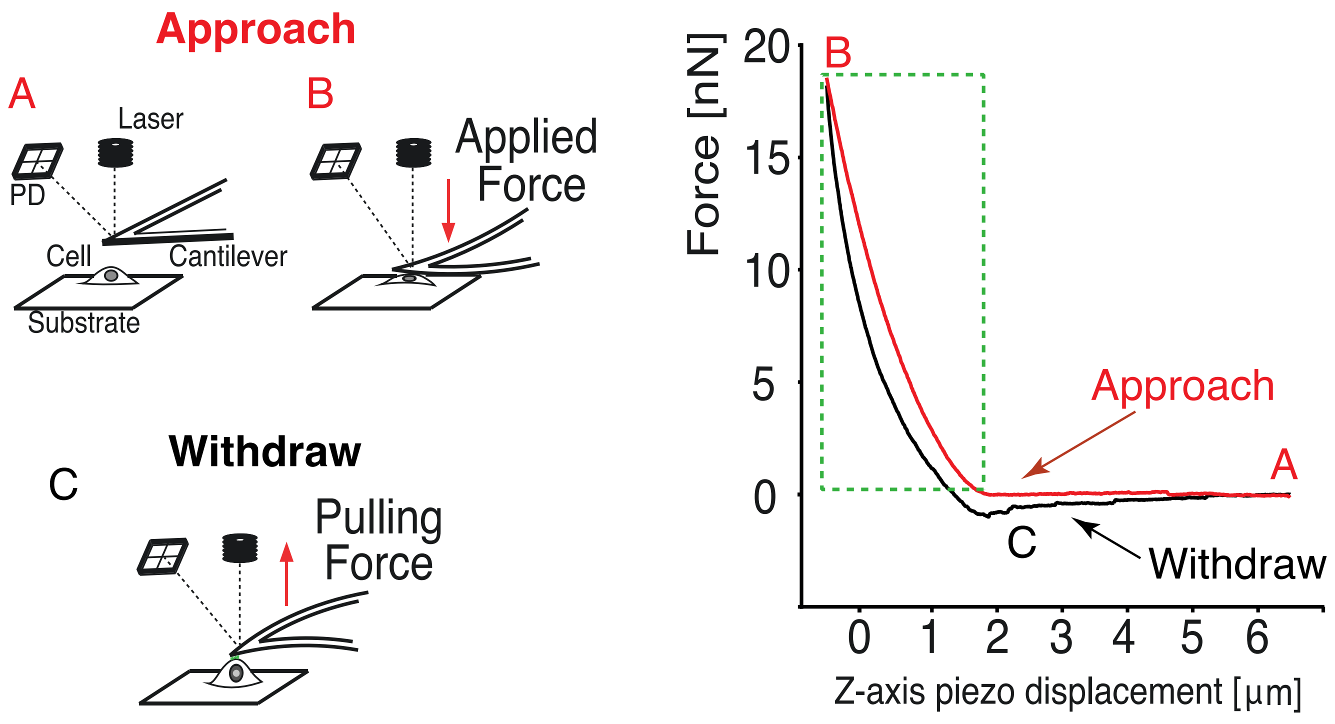

Supplementary Figure S2.

(**A**) Cartoon representative of single cell force spectroscopy by AFM. A single living cell is approached by the AFM cantilever (A), recording a force-distance curve (red curve) until the maximum cell compression is reached (B). Thereafter, the cantilever is pulled-off from the cell (C) and a withdraw curve is recorded (black curve). Hysteresis between the curves along y-axis defines the plasticity index (green dashed box).

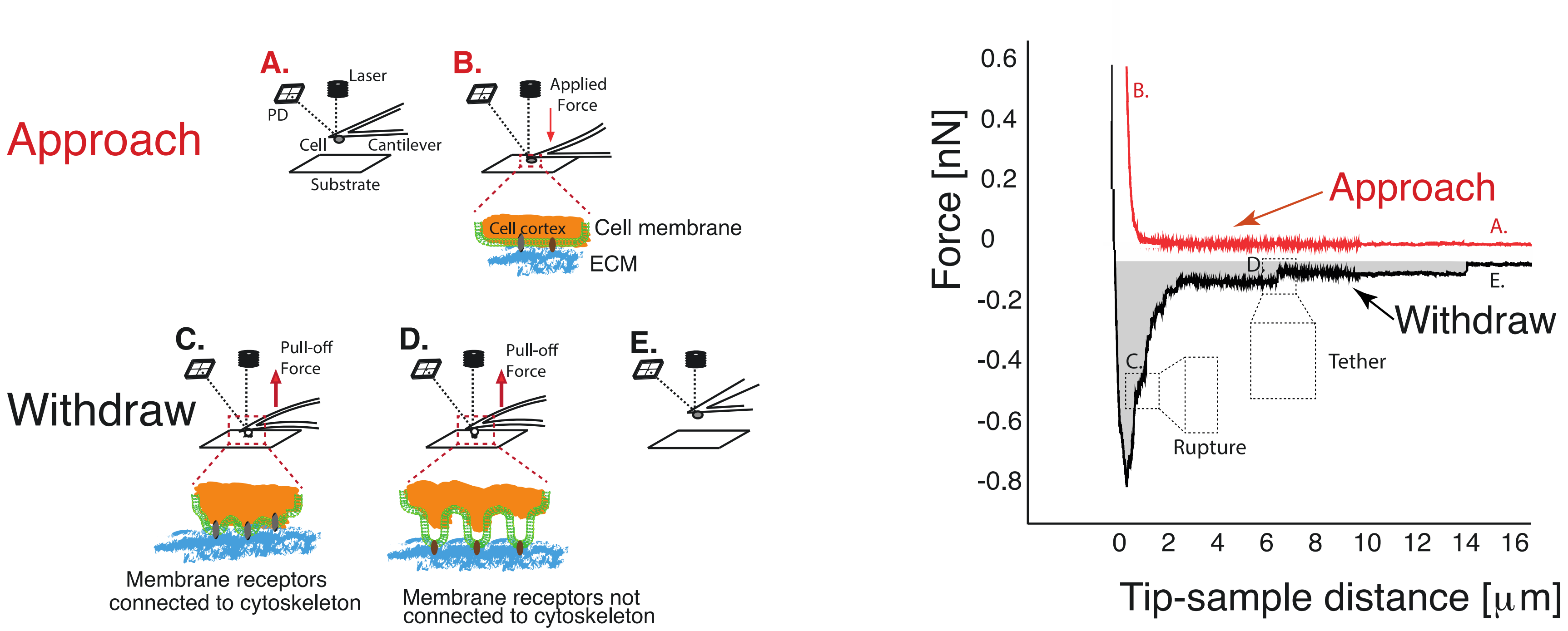

Supplementary Figure S3

(**A**) Representative cartoon of a typical cell-to-ECM interaction measurement by AFM. In the approaching phase (red curve), a cell is captured, attached and let adhere to a fibronectin functionalized cantilever (A); next, cell is pressed with a constant force, over a surface coated with the ECM protein of interest, to establish a firm interaction between the cell and the substrate (B). Thereafter, the cell is pulled-off (withdraw phase – black curve) and the interactions between ECM and the cell membrane adhesion receptors linked (ruptures – C) or not linked (tethers – D) to the cytoskeleton are recorded (E). The energy required to detach each cell from the specific substrate (work of detachment – grey area) is also recorded.
